# Evaluating Co-Designed vs Researcher-Driven Personalized Feedback Formats in a Brief Digital Alcohol Use Intervention: Mixed Methods Study

**DOI:** 10.2196/87393

**Published:** 2026-06-12

**Authors:** Antoinette Poulton, Joanna Bowen, Gezelle Dali, Cindy Chew, Katrina Prior, Emma K Devine, Louise Birrell, Lexine A Stapinski, Robert Hester

**Affiliations:** 1 Melbourne School of Psychological Sciences The University of Melbourne Parkville, Victoria Australia; 2 The Matilda Centre for Research in Mental Health and Substance Use The University of Sydney Sydney, New South Wales Australia

**Keywords:** alcohol, Capacity, Opportunity, Motivation–Behavior, co-design, COM-B, community-based participatory research, digital health, mixed methods design, personalized intervention

## Abstract

**Background:**

Increasingly, brief digital interventions are being implemented to combat alcohol misuse. While the utility of incorporating personalized feedback into these interventions has been explored, less work has concentrated on how consumer-driven research might be harnessed to better tailor personalized feedback. Critically, the relative efficacy of co-designed interventions vs those designed by research teams without stakeholder input has not typically been explored.

**Objective:**

This study aimed to use an exploratory sequential mixed methods approach to elicit user-designed personalized feedback to incorporate into an existing brief digital intervention developed to combat hazardous drinking; investigate differences in capacity, opportunity, and motivation (as per the Capacity, Opportunity, Motivation–Behavior model) to alter alcohol use behavior; examine whether participants prefer the co-designed product over those designed by research teams; and evaluate change in actual consumption over time as a function of co-designed (image-based) vs researcher-designed (image- or text-based) personalized feedback.

**Methods:**

Part 1 used an online co-design process involving a convenience sample of young adult focus group participants (N=40; mean age 21.87, SD 7.51 years). Part 2 surveyed a convenience sample of young adults who drank alcohol regularly (N=222; mean age 21.14, SD 4.69 years). Participants were assigned to 1 of 3 personalized feedback conditions (researcher designed, text-based; researcher-designed, image-based; and co-designed, image-based) using block allocation with stratification based on alcohol intake and impulsivity level. Feedback was delivered via email, and impact on capacity, opportunity, and motivation to alter alcohol use behavior, plus preferred format of feedback, was examined (10.76, SD 14.05 days) via mixed and 1-way ANOVAs and post hoc tests. Change in alcohol consumption (89.70, SD 21.59 days) was investigated via a series of repeated-measures ANOVA and post hoc tests.

**Results:**

In Part 1, qualitative thematic analysis of focus group data identified 6 overarching themes: visual representation, clarity, accessibility, brevity, peer comparison, and harm. Following this analysis, personalized feedback regarding alcohol intake and brain health was available in 3 formats: researcher designed, text-based; researcher-designed, image-based; and co-designed, image-based. In Part 2, there were no differences in capacity, opportunity, and motivation to alter alcohol use behavior as a function of feedback format (η_p_^2^=0.01). Participants preferred co-designed, image-based feedback over researcher-designed, image-based (*d*=0.55) or researcher designed, text-based (*d*=0.83) feedback. Regardless of feedback format, all participants significantly reduced total alcohol intake (η_p_^2^=0.11) and alcohol-related harm (η_p_^2^=0.05). Participants in image-based conditions (researcher-designed or co-designed) reduced frequency of drinking to a greater extent (*d*=0.56) than those in the researcher-designed, text-based feedback condition (*d*=0.39).

**Conclusions:**

Though the principles of co-design are undeniably valuable, the efficacy of co-designed interventions, over and above those designed by research teams without user input, requires further research.

**Trial Registration:**

OSF Registries osf.io/rnbv8; https://osf.io/y4bhs

## Introduction

Alcohol misuse is a major global health concern. Worldwide, 4.7% of deaths are attributable to alcohol consumption, and 115.9 million disability-adjusted life years are lost [1]. Although tangible costs—such as alcohol-related health care and court expenses—are vast, the intangible costs of alcohol misuse are even greater, encompassing widespread impacts on individuals, their families, friends, and victims of alcohol-related assaults and other crimes [2]. As such, hazardous drinking—defined by the World Health Organization (WHO) as consumption that renders individuals at risk of injury and potentially harmful consequences [1]—is often the target of research. A substantial literature has sought to capitalize on the power of brief digital interventions designed to reduce hazardous drinking [3-5]. Although the efficacy of incorporating personalized feedback into these interventions has been explored [4,6], an underinvestigated area is how consumer-driven research may be harnessed to better shape personalized feedback and the impact of such tailoring.

Consumer-driven co-design research has become a priority worldwide, including in Australia [7-9]. Co-design encompasses practices ranging from facilitated focus groups to collaborative prototyping [10] and aims to engage a diverse pool of people in the design of tools for their own use [11]. By treating people as experts on themselves and their experiences [12], co-design offers an ethical and pragmatic approach to improving digital health services, especially for youth [13-17]. Evidence demonstrates that co-designed products are well-received [18-20] and yield improvements in health literacy [21], awareness of health risks [22], and symptoms of depression [23]. Co-designed interventions targeting alcohol use in adolescents have also been shown to reduce consumption and harm [24]. Nonetheless, none of these studies provide evidence that co-designed products or interventions deliver superior outcomes compared with products designed without stakeholder input.

While some researchers have highlighted the general lack of product and intervention testing in co-design research [11,12,25], there is a particularly salient gap related to investigations that directly compare co-designed products or interventions with those developed without stakeholder input [26]. For instance, a systematic review of co-designed interventions for anxiety and depression found no study compared the effectiveness of co-designed interventions against interventions not developed through co-design [16]. Although identifying a suitable comparison product may be challenging, it is feasible. For example, Thabrew et al [27] found their co-designed app significantly reduced anxiety levels in young users over an 11-week period, with sustained improvement at 3-month follow-up. If researchers had added a comparison group that used a researcher-designed anxiety reduction app, of which there are many [28,29], this would have contributed to the research gap around the relative effectiveness of co-designed interventions vs those designed without stakeholder input.

One way to test the relative efficacy of co-designed products against those developed by researchers would be to use the Capacity, Opportunity, Motivation–Behavior (COM-B) model [30,31]. This framework assesses the impact of a behavior change intervention as a function of an individual’s knowledge and skills (capacity), their resources to enact change (opportunity), and the reflective and automatic processes related to planning, decision-making, impulse control, and emotion regulation that underpin motivation [30]. This model has been used to identify barriers and facilitators associated with implementing screening and brief interventions for alcohol use in primary care [32,33], to examine the impact of drinking guidelines on alcohol intake [34], and to explore factors influencing drinking behavior in young Australian adults [35]. By conceptualizing behavior change as a dynamic system, the COM-B model supports a comprehensive assessment of the likelihood of behavior change following a brief digital intervention.

This exploratory sequential mixed methods study aims to use co-design to generate user-designed feedback to incorporate into an existing brief digital intervention developed to combat hazardous drinking [6]. We hypothesize that participants who receive co-designed feedback will score more highly on the COM-B survey relative to those who receive researcher-designed feedback. We also hypothesize that participants will rate the co-designed feedback as more appealing than researcher-designed feedback. In keeping with our previous findings, we additionally hypothesize that individuals will reduce their alcohol intake over time in response to personalized feedback [6]. We are, however, agnostic regarding whether participants who receive co-designed feedback will decrease their drinking to a significantly greater extent than those who receive researcher-designed feedback. Thus, we expect not only to improve our existing intervention but to simultaneously address the research gap regarding the relative efficacy of co-designed health interventions vs those designed without stakeholder input.

## Methods

### Overview

This study builds on an existing intervention, which has already been shown to reduce alcohol intake by 32% to 35% in hazardous drinkers [6]. The original intervention involved emailing participants personalized researcher-designed, text-based feedback about their alcohol consumption and brain health (especially impulsivity). In Part 1 of this study, we sought the input of stakeholders to improve the appeal, comprehensibility, and effectiveness of the feedback. In Part 2, we investigated the efficacy of the co-designed product, using the COM-B model to examine capacity, opportunity, and motivation to alter alcohol intake behavior. We also examined the appeal of this product and considered the relative impact on subsequent alcohol intake behavior.

### Part 1: Co-Design

#### Preparation for Co-Design Focus Groups

**Figure 1:**
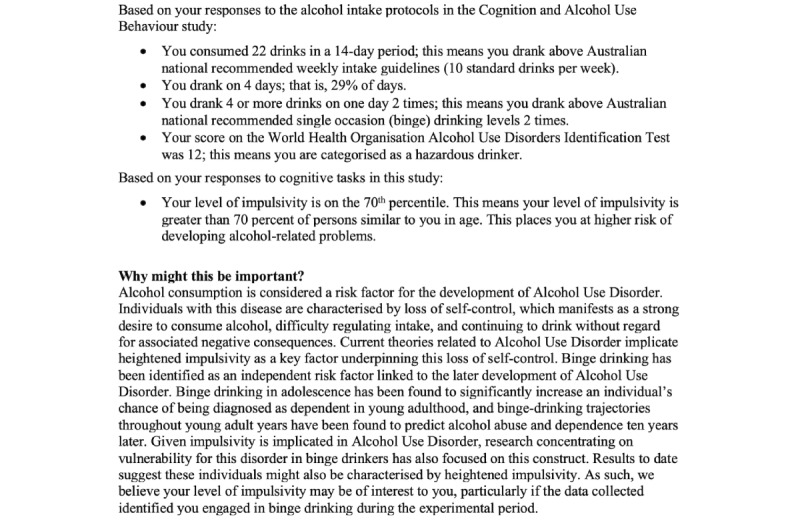
Researcher-designed, text-based personalized feedback (derived from the pilot study) used in Part 1 as a prompt for focus group discussions and in Part 2 to represent text-based feedback developed by researchers without user input.

To maximize youth involvement in the co-design process, the Youth Advisory Board at the Matilda Centre for Research in Mental Health and Substance Use (University of Sydney, Australia) was consulted for advice on running co-design focus groups that are accessible and inclusive. The Youth Advisory Board is comprised of a culturally, linguistically, and gender-diverse group of 10 members aged 16 to 25 years with representatives from metropolitan, rural, regional, and remote areas of Australia [36]. Members bring expertise and an interest in mental health and substance use via their own lived experience (personal or as carers). The Board advised on the design of materials and questions for use in focus group sessions. Members suggested that focus groups be presented with an overview of the overarching research, the original researcher-designed, text-based personalized feedback used in the pilot study (Figure 1), plus other examples of individualized feedback (Figure S1 in Multimedia Appendix 1), all as a springboard for further discussion. Thus, the researcher-designed, image-based feedback was conceived to show how personalized feedback information might be presented more graphically. This feedback was later used in Part 2 of the study to represent the type of image-based feedback that would have been developed by researchers without user input.

#### Focus Group Participants

A convenience sample of 40 young Australian adult participants was recruited via social media posts, advertisements on the University of Melbourne student portal, sporting clubs, and researcher networks. Beyond a minimum age of 17 years, there were no other exclusion criteria.

#### Focus Groups Procedure

**Figure 2:**
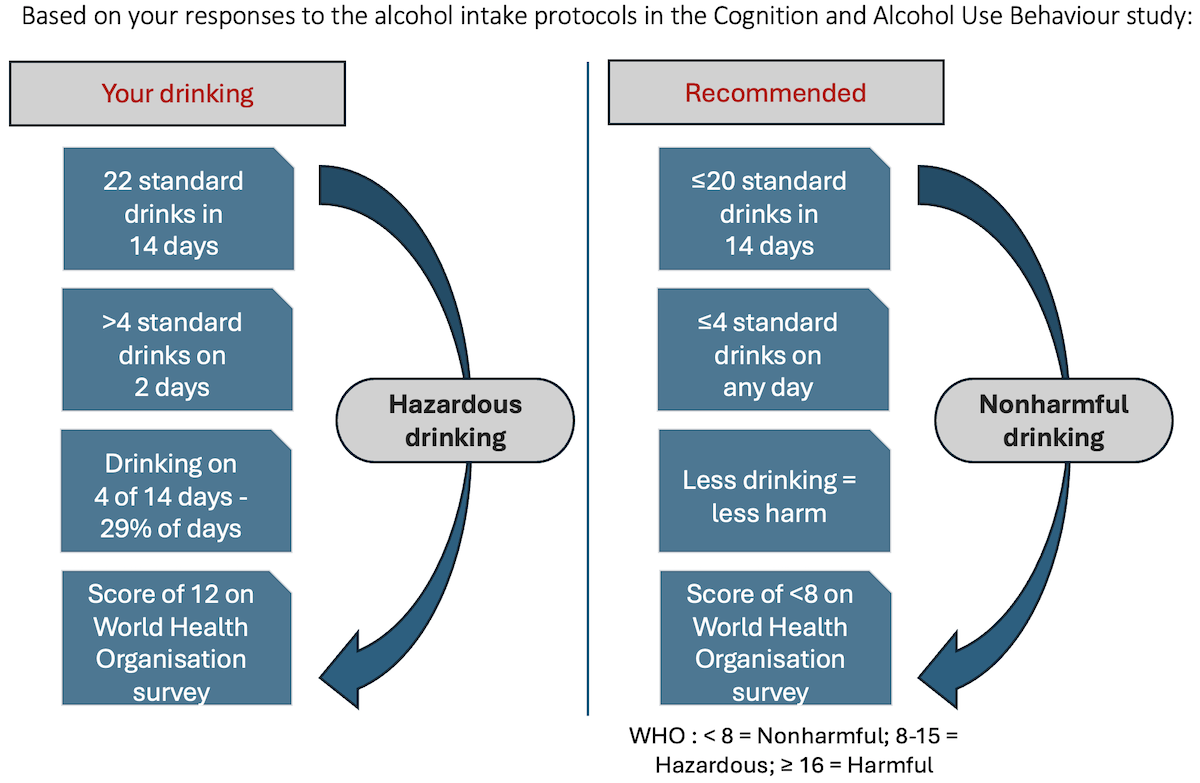
Researcher-designed, image-based feedback about alcohol intake (developed following consultation with the Youth Advisory Board at the Matilda Centre for Research in Mental Health and Substance Use) used in Part 1 as a prompt for focus group discussions and in Part 2 to determine the efficacy of the feedback.

**Figure 3:**
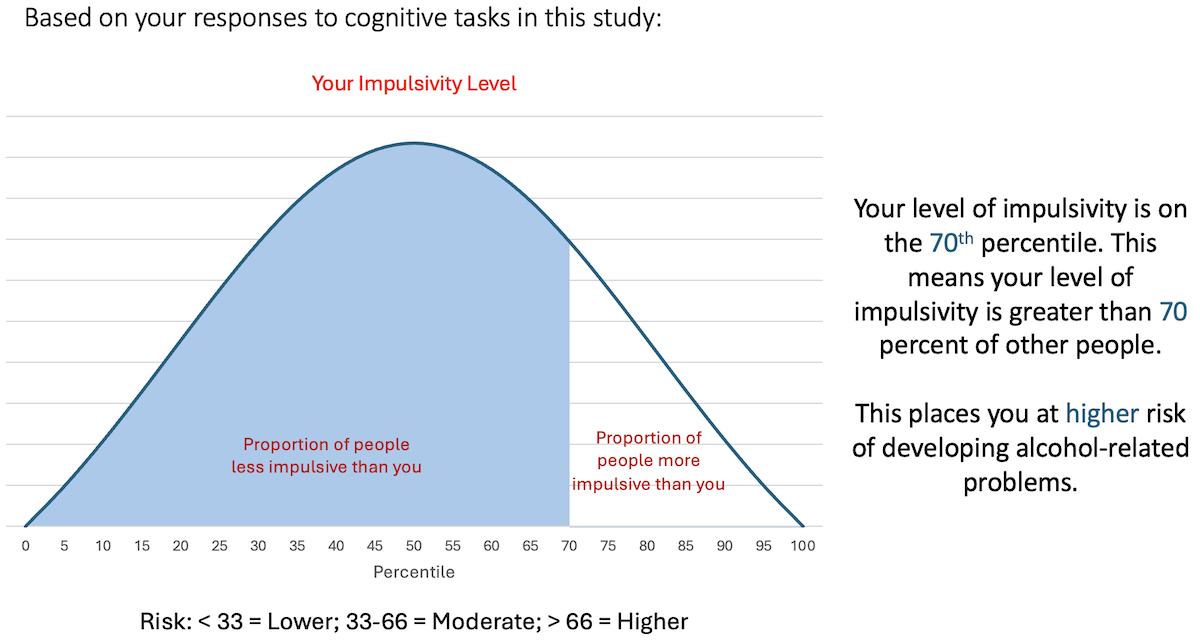
Researcher-designed, image-based feedback about brain health and impulsivity (developed following consultation with the Youth Advisory Board at the Matilda Centre for Research in Mental Health and Substance Use) used in Part 1 as a prompt for focus group discussions and in Part 2 to determine the efficacy of the feedback.

**Figure 4:**
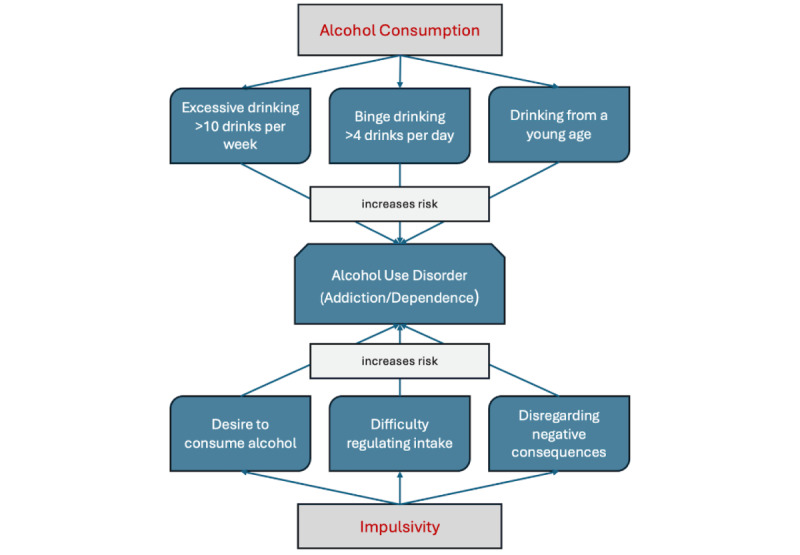
Researcher-designed, image-based feedback with general information (developed following consultation with the Youth Advisory Board at the Matilda Centre for Research in Mental Health and Substance Use) used in Part 1 as a prompt for focus group discussions and in Part 2 to determine the efficacy of the feedback.

To promote youth attendance, focus groups were held online and scheduled outside of school and university hours. Groups were organized to include attendees of similar ages to ensure that younger participants were comfortable speaking up without concerns about older participants’ perceptions or approval. When presenting to the focus groups, researchers used accessible language and paused regularly to encourage participants to ask questions and offer input. Following an overview of the broader project, the presenting researcher used semistructured questions to prompt discussion but also allowed participants to direct the dialogue where possible. Drawing on the procedure outlined by Debenham et al [22], the presenter stated there were no wrong answers, or stupid questions, nor would criticism of the feedback examples be taken personally. The goal was to create a safe, nonjudgmental, and collaborative atmosphere. So that participants were true partners in producing the co-designed feedback, when opinions differed, the researcher sought consensus about the ideal way to present each component of the feedback [12]. Figure 1 displays the original researcher-designed, text-based personalized feedback used in the pilot study [6]. While we sought general input about the appearance and presentation of the feedback, we also asked for specific detail about how to break down the original text into (1) information about alcohol intake; (2) information about brain health, specifically impulsivity; and (3) general information about the links between impulsivity and alcohol misuse. Figures 2-4 shows some of the springboard examples used in focus groups. Multimedia Appendix 1 contains all examples.

#### Qualitative Analyses

Focus groups were recorded via Zoom (version 6.4.0; Zoom Communications, Inc) and transcribed (by JB). The transcripts were analyzed using thematic analysis, a flexible and foundational approach to qualitative investigations [37]. Thematic analysis can be either inductive or deductive; this study took a strongly inductive, data-driven approach, focusing on what the data said rather than on a priori theories or conclusions [38]. Thematic analysis has proven useful in other participatory design research [21,25,39].

Following the key steps of thematic analysis, 2 researchers (AP and JB) independently familiarized themselves with the transcripts, then each generated an initial list of codes, which became a preliminary list of themes. The researchers then collaborated to negotiate a core list of themes, which a third researcher (CC) reviewed against the original transcripts. A final meeting was held with all 3 researchers, resulting in a consensus list of themes and feedback. The COREQ (Consolidated Criteria for Reporting Qualitative Research) checklist can be found in Table S1 in Multimedia Appendix 1.

### Part 2: Efficacy

#### Participants

A convenience sample of 222 young Australian adults who drank alcohol regularly were recruited via social media posts, advertisements on the University of Melbourne student portal, and researcher networks. Eligibility criteria specified that they must be aged 14 years or older and that they drink alcohol at least weekly.

#### Procedure

At T1 (baseline), participants completed an online survey comprising demographic questions, measures of alcohol consumption, anxiety and depression, and cognitive tasks assessing impulsivity. At the completion of T1, participants were allocated to 1 of 3 feedback conditions (original researcher-designed, text-based; researcher-designed, image-based; or co-designed, image-based), with stratification based on impulsivity and alcohol intake levels [6]. Impulsivity was derived from performance on cognitive tasks using normative data [40] and categorized as high, medium or low impulsivity via a tertile split. Alcohol intake was categorized as nonharmful, hazardous, or harmful using Alcohol Use Disorders Identification Test (AUDIT) scores [41,42].

To ensure timely feedback while maintaining stratification, we used dynamic allocation: as T1 data became available, participants were assigned a code (by AP) representing their impulsivity and alcohol category (9 possible combinations). They were then allocated (by JB) to whichever feedback condition had the lowest count in that category at the time, ensuring balanced representation across conditions. The allocation code was concealed from the other authors. Participants were not informed there were different feedback conditions in this study, nor were they explicitly informed which condition they had been allocated to.

**Figure 5:**
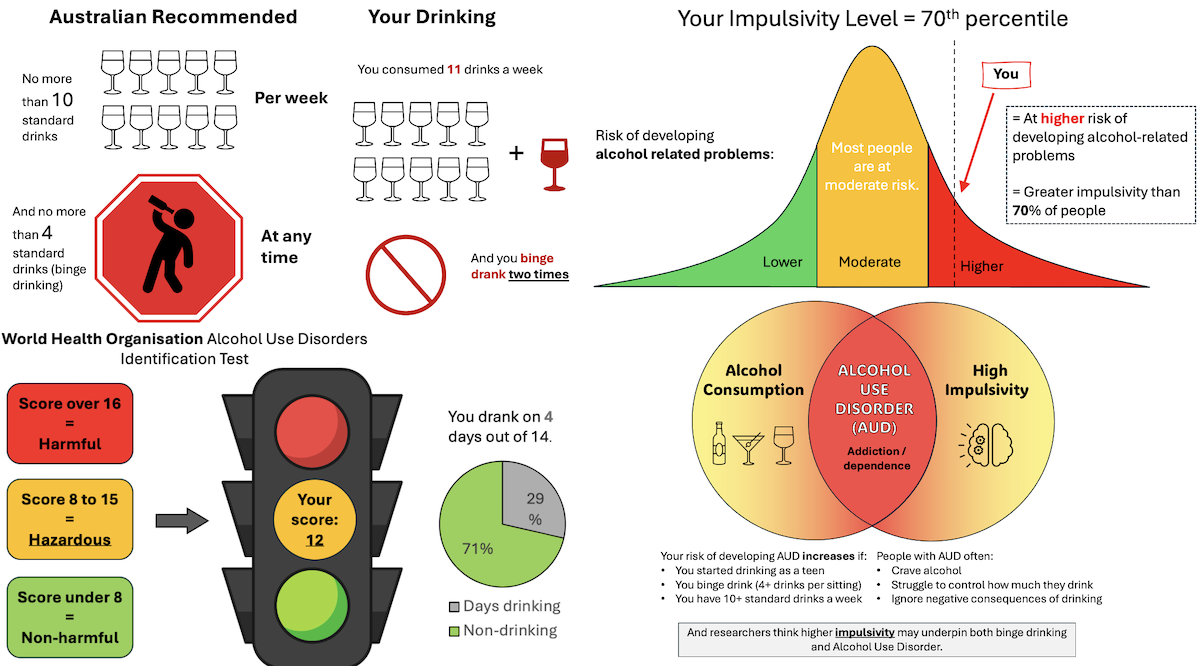
Co-designed, image-based feedback (developed following consultation with focus groups) and used in Part 2 to determine the efficacy of the feedback.

Participants were emailed personalized feedback related to alcohol intake, impulsivity, and general information about the links between brain health (especially impulsivity) and alcohol misuse. For those assigned to the original researcher-designed, text-based feedback condition, this feedback was entirely text-based (Figure 1). Those assigned to the researcher-designed, image-based feedback condition were sent feedback identified by focus groups as the best of the examples developed by researchers without user input (Figures 2-4). Participants in the co-designed feedback condition were sent image-based feedback developed following the recommendations of focus groups from Part 1 of this study (Figure 5).

At T2, participants were invited to complete a survey that included a question about how honestly they had reported alcohol use at T1, as well as questions related to capacity, opportunity, and motivation to alter their drinking behavior (COM-B). They were then shown all 3 examples of the feedback in counterbalanced order (using Qualtrics counterbalancing code) and asked questions related to preferred format. They were not provided with information about how each type of feedback had been generated, and the feedback contained no personalized information related to alcohol intake or impulsivity.

At T3, participants were invited to again complete surveys of alcohol consumption and cognitive tasks measuring impulsivity.

### Measures

#### Monetary Choice Questionnaire

The monetary choice questionnaire (MCQ) is a measure of choice impulsivity, or the propensity to choose immediately rewarding options regardless of delayed consequences. Participants make hypothetical choices between 27 small immediately available monetary rewards and larger rewards obtainable only after some delay [43]. Responses can be expressed as a hyperbolic function. The extent to which an individual discounts a delayed reward is measured by the slope of the function, or overall *k*; larger *k* values indicate greater discounting of the delayed reward, or heightened choice impulsivity [44]. Consistency scores are calculated to capture whether responses are consistent with previous selections; participants with consistency scores below 75% are excluded [44].

#### Stop Signal Task

The stop signal task (SST) is a widely used measure of inhibitory control that captures the capacity to inhibit a response or reaction, especially once that response has been initiated. Thus, it is a useful measure of how well individuals can control their impulsivity [45,46]. The task, which has been fully described in previous work, consists of a practice block of 32 trials and 3 blocks of 64 experimental trials [46]. The main variable of interest is stop signal reaction time (SSRT), with higher values indicative of greater impulsivity. Participants are excluded if stop accuracy is less than 25% or greater than 75%, go accuracy is less than 60%, go errors are greater than 10%, SSRT is less than 50 ms, or reaction time (RT) on unsuccessful stops (choice errors and premature responses) is greater than RT on go trials [47,48].

#### AUDIT Measure

The 10-item AUDIT assesses alcohol intake, alcohol-related problems, and alcohol dependence over the preceding 6 months [42]. Scores suggest nonharmful (<8), hazardous (8-15), or harmful (≥16) alcohol use [41].

#### Timeline Followback

Timeline Followback is a retrospective diary method of collecting alcohol consumption information [49,50]. Participants record the number of standard drinks consumed over the preceding 14 days.

#### Generalized Anxiety Disorder Scale

The 7-item Generalized Anxiety Disorder-7 (GAD-7) assesses symptoms of anxiety over the previous 2 weeks; scores ≥10 suggest moderate anxiety [51,52].

#### Patient Health Questionnaire

The 9-item Patient Health Questionnaire-9 (PHQ-9) assesses depressive symptoms over the previous 2 weeks; scores ≥10 indicate moderate depression [53,54].

#### Advanced Progressive Matrices

A 6-item abridged online untimed version of the Advanced Progressive Matrices (APM) was used to assess intellectual capacity. The APM-6 forms half of the APM-12U, an abridged online untimed 12-item version of the APM [55,56].

#### COM-B: Alcohol Intake Questionnaire (COM-B:AIQ)

Previous research has successfully used the COM-B model to explore alcohol-related behavior change [34]. Working from West and Michie’s guide [30], the 19-item COM-B: Alcohol Intake Questionnaire (COM-B:AIQ) was developed to measure capacity, opportunity, and motivation to alter alcohol intake behavior following the brief intervention under investigation. Nine items assess capability (knowledge, skills, and self-efficacy), 5 items measure opportunity (social and environmental), while a further 5 items assess motivation (reflective and automatic). A higher score on the COM-B:AIQ indicates a greater likelihood of behavior change. The full measure is available in Table S2 in Multimedia Appendix 1.

#### Preferred Feedback

Participants rated whether they liked the “look and feel” of the feedback, how easy it was to understand, and how well they thought they would remember the information. Responses were provided via a Likert scale (1=strongly disagree to 6=strongly agree). Higher scores indicate greater liking.

### Quantitative Analyses

At T1 (baseline), a percentile score was assigned to each participant for performance on each of the MCQ and SST [6]. The mean of these scores formed the average impulsivity index for each participant. Regarding the MCQ, hyperbolic *k* values were log transformed to correct for nonnormal distribution. Untransformed values (3 decimal places) are displayed in tables to assist with interpretation. With the SST, SSRT was calculated according to the integration method [48]. This involves arranging RTs on go trials (correct, incorrect, and omissions) in ascending order, with the maximum correct go RT value being substituted in the case of any go omissions. Then, the nth RT is identified by multiplying the proportion of unsuccessful stops by the number of go trials. SSRTintegration is calculated by subtracting mean stop signal delay from this nth RT. Due to SST exclusions at T1 (n=19) and T3 (n=9), T3 analyses involving change in impulsivity involved 78 participants. There were no MCQ exclusions.

We conducted 1-way ANOVA and chi-square analyses to determine whether there were any demographic, alcohol intake or cognitive task performance differences as a function of feedback group allocation at T1. We also used 1-way ANOVA to consider whether there were any differences in capacity, opportunity, and motivation to change behavior as a function of feedback group allocation at T2. A mixed measures ANOVA was used at T2 to examine rating or liking of the feedback types and to check whether preferences were influenced by T1 feedback group allocation. Feedback type (original researcher-designed, text-based; researcher-designed, image-based; and co-designed, image-based) formed the within-subjects factor, while T1 feedback group allocation (original researcher-designed, text-based; researcher-designed, image-based; and co-designed, image-based) formed the between-subjects factor. A series of repeated measures ANOVA were used to investigate change in alcohol use behavior (AUDIT score, total standard drinks, frequency of drinking, and occasions when ≥4 drinks were consumed in 1 episode) and impulsivity from T1 to T3 as a function of feedback group allocation. Time (T1 and T3) formed the within-subjects factor, while feedback group allocation (original researcher-designed, text-based; researcher-designed, image-based; and co-designed, image-based) formed the between-subjects factor. Pairwise comparisons were Bonferroni-corrected.

In the case of ANOVAs, effect sizes were calculated using partial eta squared (η_p_^2^): 0.01=small, 0.06=moderate, and 0.14=large effect [57]. Cohen *d* was used as a measure of effect for post hoc tests: 0.20=small, 0.50=medium, and 0.80=large [58]. For chi-square, Cramer V was used as a measure of effect: 0.07=small, 0.21=medium, and 0.35=large effect [58]. Based on our pilot study [6], we were aware attrition from T1 to T2 would be approximately 30% to 35% but were uncertain about attrition at T3. We therefore conducted an a priori power analysis for T2 and a post hoc analysis for T3. For analyses at T2, the a priori analysis conducted using G*Power (effect size f=0.25; α=.05; power=0.80) showed a total sample of 159 was required. For analyses at T3, the post hoc analysis (effect size f=0.25; α=.05; n=91; number of measurements=2; number of groups=3) showed that power was 0.68. The CONSORT (Consolidated Standard of Reporting Trials) checklist is available in Table S3 in Multimedia Appendix 1.

### Ethical Considerations

Study protocols were reviewed and approved by the University of Melbourne Office of Research Ethics and Integrity Human Ethics Committee (approval number 26716). In both Parts 1 and 2 of this study, participants received dedicated ethics-approved plain language statements. If they decided to continue with the study, they then signed dedicated ethics-approved consent forms. Participants from Part 1 were reimbursed AU $75 (US $54) in GiftPay vouchers. Participants from Part 2 received course credit for contributing data at T1 and T2 and were reimbursed AU $15 (US $11) in GiftPay vouchers for T3. To ensure confidentiality and privacy, demographic and survey data were collected via the Qualtrics platform. Qualtrics servers are protected by high-end firewall systems. Exported datasets, audio files, and focus groups transcripts were stored separately on the University of Melbourne OneDrive cloud storage service and were accessible only to AP and JB. Participation in the study was entirely voluntary. The study was not preregistered because we sought to investigate the effectiveness of different types of feedback rather than to test the existing overarching intervention. Nonetheless, we have retrospectively registered the peer-reviewed grant proposal and outcome letter that provides a prospective description of this study [59].

## Results

### Overview

**Figure 6:**
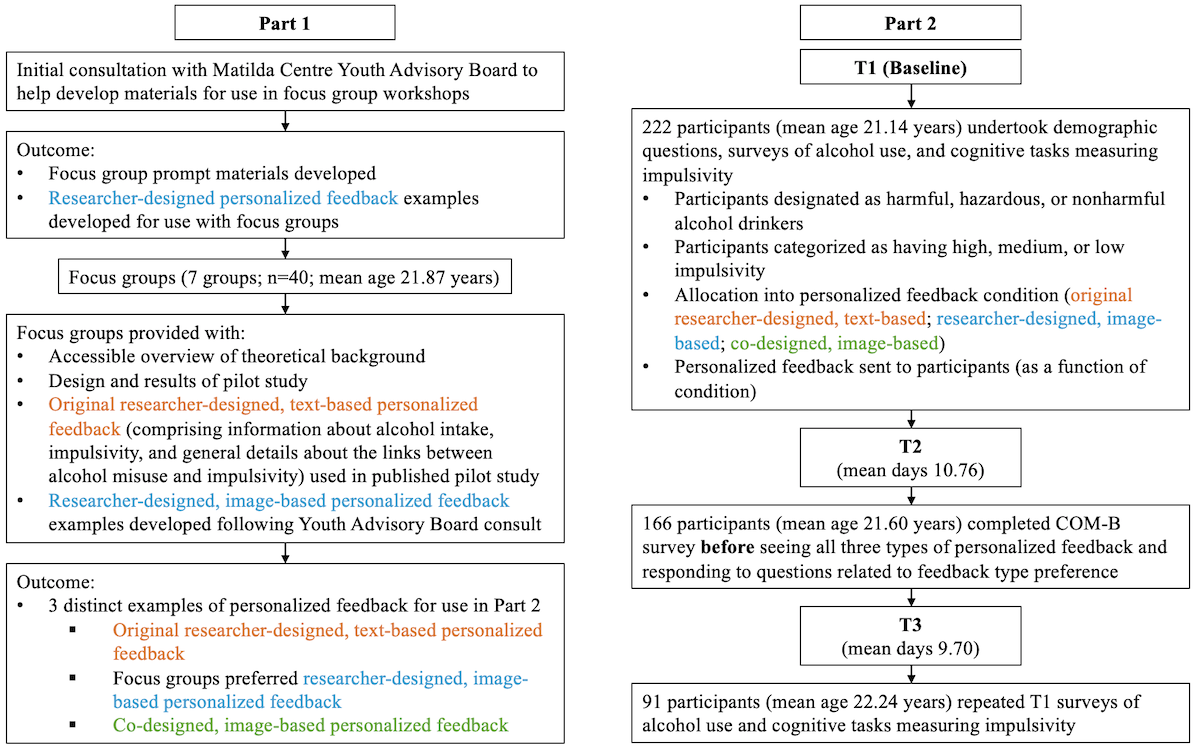
Flowchart for the mixed methods study evaluating co-designed vs researcher-driven personalized feedback formats in a brief digital alcohol use intervention. YAB: Youth Advisory Board.

**Figure 7:**
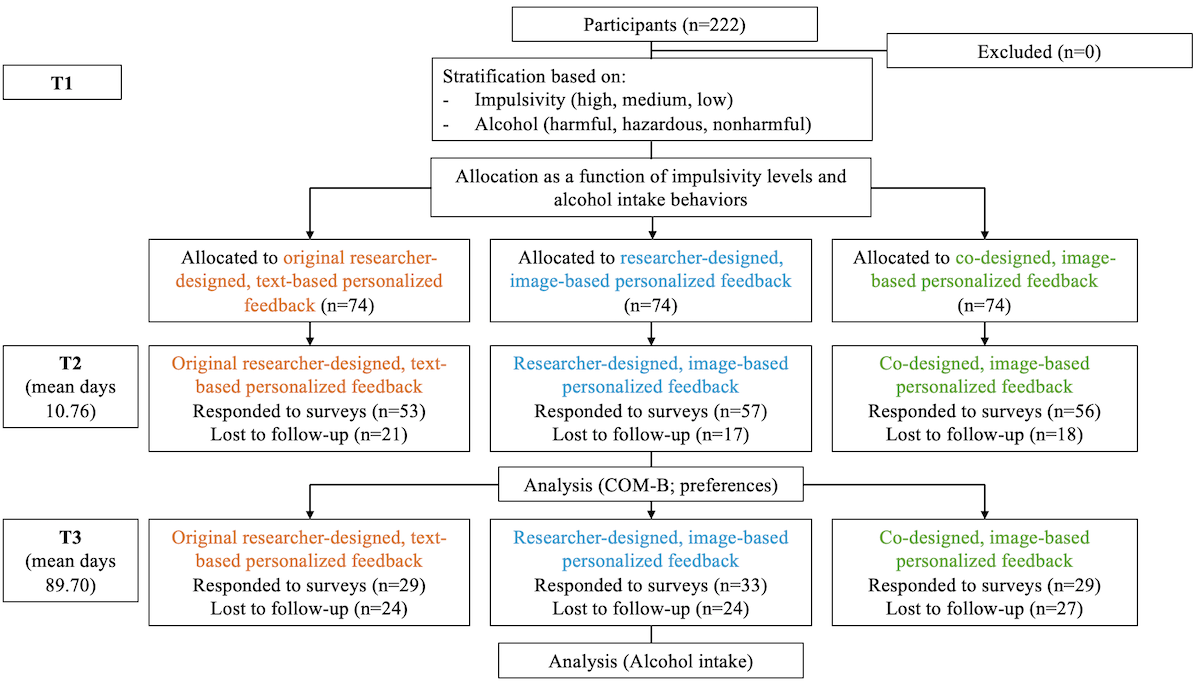
CONSORT (Consolidated Standards of Reporting Trials) flow diagram of participant enrollment in Part 2, allocation, follow-up, and analysis.

Figure 6 provides an overview of Parts 1 and 2 of this study, while Figure 7 shows the flow of participants through Part 2, including the numbers allocated to each condition across time points, those lost to follow-up, and those available for analyses.

### Part 1: Co-Design

A total of 40 participants (mean age 21.87, SD 7.51 years; range 17-47 years; 20/40, 50% female) were involved in 7 focus groups (90 minutes each). A total of 24 participants were aged <20 years, 10 were aged 20-25 years, and 6 were aged >25 years. Average years of education was 13.78 (SD 2.12) years, and the majority (34/40, 85%) hailed from capital cities or large metropolitan areas. In terms of age at first drink, 15% (6/40) had never consumed alcohol, 37.5% (15/40) had consumed their first full drink at age ≤15 years, 27.5% (11/40) at age 16 or 17 years, and 20% (8/40) at age ≥18 years.

Qualitative analysis identified 6 overarching themes, 4 of which centered on presenting information graphically. Theme 1: visual representation. This theme was best summarized as “people love infographics” and “graphics grab attention.” Theme 2: clarity. Participants stated, “visuals must be clear, simple and effective” and “a confusing graphic is as bad as a chunk of text.” Theme 3: accessibility. Participants suggested, “People are unwilling to work hard to understand something” and “communication must be easy to comprehend.” Theme 4: brevity. This was best encapsulated by “wordiness is off-putting.” The final 2 themes related to content. Theme 5: peer comparison. Participants wanted to know where they stood in relation to their peers (not the general population). A participant indicated:

I think also comparative data is quite important. I want to know how I'm comparing with the females that are my age to see if I am below average, average, because obviously, male, female, we can drink different amounts and feel different ways.

Theme 6: harm. Participants wanted access to more information about the effects of alcohol, including both long- and short-term effects. They asked for “more info about consequences of drinking small amounts regularly compared to binge drinking” and “more info on alcohol-related diseases.” The researchers identified a preconception they had brought—that youth would only be interested in the short-term effects of alcohol misuse, such as hangovers and poor academic performance. However, many young participants expressed the desire for better education about the long-term physical and mental effects of alcohol use, along with more information about the short-term effects of regular drinking below the binge-drinking threshold (≤4 drinks consumed in 1 episode, as per Australian guidelines) [60].

Researchers additionally asked for advice about how to improve the presentation of the feedback examples provided. Reactions to the original researcher-designed, text-based feedback (Figure 1) used in the pilot study [6] were uniformly negative; it was repeatedly referred to as “a chunk of text” and “dense.” Of the 3 researcher-designed, image-based feedback examples related to alcohol intake, the graphic in Figure 2 was strongly preferred across all groups (see Figure S1 in Multimedia Appendix 1 for all examples). Participants liked this graphic because they thought it was easy to navigate, well-organized, and appeared logical. Nonetheless, these researcher-designed, image-based examples were also labeled “overwhelming” by 3 participants and were generally considered “confusing.” Many participants indicated no preferences across examples, although they agreed they were better than the original researcher-designed text.

Regarding the researcher-designed, image-based feedback examples related to brain health (impulsivity), there was widespread agreement that the visual was an improvement, although most felt the original researcher-designed, text-based feedback was also easy to understand. There was consensus that the use of percentages would be better than percentiles, as participants felt that most people are more likely to understand percentages. The majority preferred the example in Figure 3 (see Figure S1 in Multimedia Appendix 1 for all examples) because it felt the most “accessible.” The researcher-designed, image-based feedback example concerning general information (Figure 4) was widely viewed as “overwhelming.” However, most participants still preferred it over the original researcher-designed text: “I think it’s much better than reading that slab of information.” While participants agreed this information was important, they were ambivalent regarding how best to present it.

Table S4 in Multimedia Appendix 1 provides full details of specific suggestions for elements to include in co-designed feedback. Co-designed, image-based feedback was developed from these suggestions, based on majority decisions by the focus groups. As such, the co-designed feedback about alcohol intake incorporated the following focus group recommendations: use graphics, such as drinking icons (especially beer glasses), as a visual representation of drinks; use red and bold text to emphasize drinking above recommended levels; use traffic-light coloring to indicate nonharmful, hazardous, and harmful drinking; use a pie chart to show frequency of drinking; use bullet-point formatting for text; and use a side-by-side layout to juxtapose personalized drinking against national recommendations. The co-designed feedback about brain health, specifically impulsivity, included the following: ensure that colors are clear and contrasting; have a “you are here” indicator with an arrow and red font; split the graph into low, medium, and high categories; color code the risk levels; and use bullet-point formatting for text. General information about the links between impulsivity and alcohol use incorporated the following: use a Venn diagram, simplify language, use colors and bold text, use bullet-point formatting, present information horizontally, and use the active voice. Figure 5 shows the co-designed, image-based feedback.

### Part 2: Efficacy

#### Descriptive Analyses

A total of 222 participants (mean age 21.14, SD 4.69 years; range 18-68 years; 176/222, 79% female) completed T1 (baseline). Of these, 166 participants (mean age 21.60, SD 5.27 years; range 18-68 years; 129/166, 78% female) completed T2 (166/222, 74.77% of the T1 sample) 11 days later, and of these, 91 (mean age 22.24, SD 6.07 years; range 18-68 years; 75/91, 82% female) returned to undertake T3 (91/166, 54.82% of the T2 sample) 3 months later. Figure 7 provides the participation flowchart.

Tables 1 and 2 show demographic statistics for participants who completed T1 (baseline) and T2 of Part 2. Demographics as a function of feedback group allocation (original researcher-designed, text-based; researcher-designed, image-based; and co-designed, image-based) are also detailed. There were no significant differences on these variables as a function of feedback group. Table 3 shows alcohol intake behavior and impulsivity at T1. There were also no significant differences in these variables as a function of feedback group.

**Table 1:** Part 2: continuous demographic statistics at T1 for all participants who completed T1 and T2, and as a function of original researcher-designed, text-based; researcher-designed, image-based; and co-designed, image-based feedback group allocation.

Variable	Total (n=166), mean (SD)	Original researcher-designed, text-based feedback (n=53), mean (SD)	Researcher-designed, image-based feedback (n=57), mean (SD)	Co-designed, image-based feedback (n=56), mean (SD)	*F* test (*df*)	*P* value	η_p_^2^
Age (years)	21.60 (5.27)	20.91 (3.76)	22.68 (7.29)	21.16 (3.75)	1.88 (2, 163)	.16	0.02
Education (years)	14.50 (1.85)	14.36 (1.81)	14.85 (1.96)	14.27 (1.73)	1.64 (2, 163)	.20	0.02
APM-6^a^	3.61 (1.65)	3.57 (1.65)	3.83 (1.66)	3.45 (1.65)	0.77 (2, 163)	.46	0.01
GAD-7^b^	6.86 (5.65)	6.83 (5.74)	6.88 (5.09)	6.86 (6.18)	<0.01 (2, 163)	>.99	<0.01
PHQ-9^c^	8.13 (6.13)	7.17 (6.07)	8.93 (6.25)	8.23 (6.06)	1.14 (2, 163)	.32	0.01

^a^APM-6: Advanced Progressive Matrices-6 (abridged 6-item version of Raven’s APM).

^b^GAD-7: Generalized Anxiety Disorder-7.

^c^PHQ-9: Patient Health Questionnaire-9.

**Table 2:** Part 2: categorical demographic statistics at T1 for all participants who completed T1 and T2, and as a function of original researcher-designed, text-based; researcher-designed, image-based; and co-designed, image-based feedback group allocation.

Variable			Total (n=166), n (%)		Original researcher-designed, text-based feedback (n=53), n (%)		Researcher-designed, image-based feedback (n=57), n (%)		Co-designed, image-based feedback (n=56); n (%)		Chi-square (*df*)			*P* value			Cramer V	
Sex ratio (male:female:other)			36 (21.7):129 (77.7):1 (0.6)		12 (22.6):41 (77.4):0 (0)		14 (24.6):43 (75.4):0 (0)		10 (17.8):45 (80.4):1 (1.8)		2.7 (4)			.61			0.09	
Country of birth												7.3 (8)			.50			0.15
	Australasia	76 (45.8)		29 (54.7)		22 (38.6)		25 (44.6)										
	Asia	76 (45.8)		19 (35.9)		32 (56.1)		25 (44.6)										
	Europe	9 (5.4)		4 (7.6)		2 (3.5)		3 (5.4)										
	Americas	4 (2.4)		1 (1.9)		1 (1.8)		2 (3.6)										
	Other	1 (0.6)		0 (-)		0 (-)		1 (1.8)										
First language												5.0 (2)			.08			0.17
	English	109 (65.7)		41 (77.4)		33 (57.9)		35 (62.5)										
	Other	57 (34.3)		12 (22.6)		24 (42.1)		21 (37.5)										

**Table 3:** Part 2: alcohol intake behavior and impulsivity at T1 for all participants who completed T1 and T2, and as a function of original researcher-designed, text-based; researcher-designed, image-based; and co-designed, image-based feedback group allocation.

Variable		Total (n=166), mean (SD)	Original researcher-designed, text-based feedback (n=53), mean (SD)	Researcher-designed, image-based feedback (n=57), mean (SD)	Co-designed, image-based feedback (n=56), mean (SD)	*F* test (*df*)	*P* value	η_p_^2^
AUDIT^a^ score		9.32 (5.12)	9.98 (5.03)	8.93 (5.25)	9.09 (5.11)	0.66 (2, 163)	.52	0.01
Timeline Followback								
	Total standard drinks	17.61 (18.60)	17.42 (13.22)	16.79 (17.65)	18.64 (23.54)	0.14 (2, 163)	.87	<0.01
	Drinking days (%)	35.09 (21.19)	32.47 (16.87)	39.18 (24.10)	33.41 (21.44)	1.65 (2, 163)	.20	0.02
	Days with ≥4 drinks	1.78 (2.30)	1.81 (1.76)	1.61 (2.32)	1.93 (2.73)	0.27 (2, 163)	.77	<0.01
MCQ^b^								
	Overall *k*	0.027 (0.047)	0.022 (0.030)	0.023 (0.042)	0.036 (0.061)	1.86 (2, 163)	.16	0.02
SST^c^								
	SSRTintegration^d^	238.70 (51.39)	237.45 (48.53)	246.64 (41.44)	231.65 (62.20)	1.09 (2, 163)	.34	0.02
	Impulsivity index	48.63 (21.82)	46.17 (22.48)	49.28 (21.25)	50.31 (22.02)	0.46 (2, 163)	.63	0.01

^a^AUDIT: Alcohol Use Disorder Identification Test.

^b^MCQ: Monetary Choice Questionnaire.

^c^SST: stop signal task.

^d^SSRTintegration: stop signal reaction time calculated using the integration method.

#### COM-B:AIQ as a Function of Feedback Group

At T2, 77.1% (128/166) and 22.9% (38/166) of the sample, respectively, indicated that they had been either “completely honest” or “mostly honest” when responding to T1 questions about alcohol consumption. No participants selected the “mostly not honest” or “not at all honest” options.

Table 4 shows scores for each of capacity, opportunity, and motivation for behavior change, as well as total COM-B:AIQ score, both for the total sample and as a function of feedback group allocation. There were no differences between groups on total COM-B:AIQ score, *F*_2,163_=0.77; *P*=.47; η_p_^2^=0.01, or on subsections related to capacity, *F*_2,163_=0.73; *P*=.49; η_p_^2^=0.01, opportunity, *F*_2,163_=0.68; *P*=.51; η_p_^2^=0.01, or motivation, *F*_2,163_=0.61; *P*=.55; η_p_^2^=0.01.

**Table 4:** Part 2: Capacity, Opportunity, Motivation–Behavior: Alcohol Intake Questionnaire (COM-B:AIQ) scores at T2 for all participants who completed T1 and T2, and as a function of original researcher-designed, text-based; researcher-designed, image-based; and co-designed, image-based feedback group allocation.

COM-B:AIQ component	Total (n=166), mean (SD)	Original researcher-designed, text-based feedback (n=53), mean (SD)	Researcher-designed, image-based feedback (n=57), mean (SD)	Co-designed, image-based feedback (n=56), mean (SD)
Capacity	27.31 (3.48)	27.76 (3.40)	26.97 (2.93)	27.23 (14.04)
Opportunity	22.37 (3.73)	22.62 (3.42)	22.60 (3.48)	21.89 (4.24)
Motivation	20.63 (3.79)	20.68 (3.44)	21.00 (3.65)	20.21 (4.26)
COM-B^a^ total	70.31 (7.47)	71.06 (6.25)	70.56 (6.78)	69.34 (9.07)

^a^COM-B: Capacity, Opportunity, Motivation–Behavior.

To further interrogate responses on the COM-B:AIQ, we considered how participants answered questions specifically addressing Australian national drinking guidelines [60]. In total, 23.5% (39/166) of the sample correctly answered the question probing how many total standard drinks could safely be consumed per week (10 drinks). There was no difference in correct responses as a function of feedback group allocation, *χ*^2^_2_=1.2; *P*=.55; V=0.09. Only 15.7% (26/166) correctly answered the question regarding safe daily consumption (≤4 drinks). Again, there was no difference in correct responses as a function of feedback group allocation, *χ*^2^_2_=2.3; *P*=.31; V=0.12. As focus group participants had specifically indicated percentiles might be difficult for people to interpret, we also examined responses to the COM-B:AIQ question ascertaining knowledge around percentiles. In all, 59% (98/166) of the sample answered the question correctly. There was no difference in correct responses as a function of feedback group allocation, *χ*^2^_2_=2.3; *P*=.32; V=0.12.

#### Preferred Feedback Format as a Function of Feedback Group

Participants undertook T2 testing 10.76 (SD 14.05) days after completing T1 (and following delivery of feedback). Table 5 details rating, or liking, of each feedback type, both for the whole sample and as a function of feedback group allocation. A mixed measures ANOVA showed a significant main effect of feedback type, *F*_2,326_=38.11; *P*<.001; η_p_^2^=0.19. Pairwise comparisons revealed participants preferred co-designed, image-based over the researcher-designed, image-based (*P*<.001; *d*=0.55) and original researcher-designed, text-based (*P*<.001; *d*=0.83) feedback. There was no main effect of feedback group allocation, *F*_2,163_=2.18; *P*=.12; η_p_^2^=0.03, and no interaction between rating and feedback group, *F*_4,326_=1.84; *P*=.12; η_p_^2^=0.02.

**Table 5:** Part 2: preferred feedback format at T2 for all participants who completed T1 and T2, and as a function of original researcher-designed, text-based; researcher-designed, image-based, and co-designed, image-based feedback group allocation.

Feedback format	Total (n=166), mean (SD)	Original researcher-designed, text-based feedback (n=53), mean (SD)	Researcher-designed, image-based feedback (n=57), mean (SD)	Co-designed, image-based feedback (n=56), mean (SD)
Original text-based	20.19 (5.80)	20.13 (5.38)	21.04 (5.62)	19.39 (6.32)
Researcher-designed image-based	21.69 (5.62)	20.81 (5.87)	23.09 (4.97)	21.11 (5.83)
Co-designed	24.65 (4.66)	23.62 (5.82)	24.77 (4.26)	25.48 (3.58)

#### Change in Alcohol Consumption as a Function of Feedback Group

Participants undertook T3 testing 89.70 (SD 21.59) days after completing T2. In terms of return rate, 54.72% (29/53) of those allocated to the original researcher-designed, text-based feedback group undertook T3 testing, 57.89% (33/57) of those allocated to the researcher-designed, image-based group, and 51.79% (29/56) of those allocated to the co-designed, image-based feedback group.

**Figure 8:**
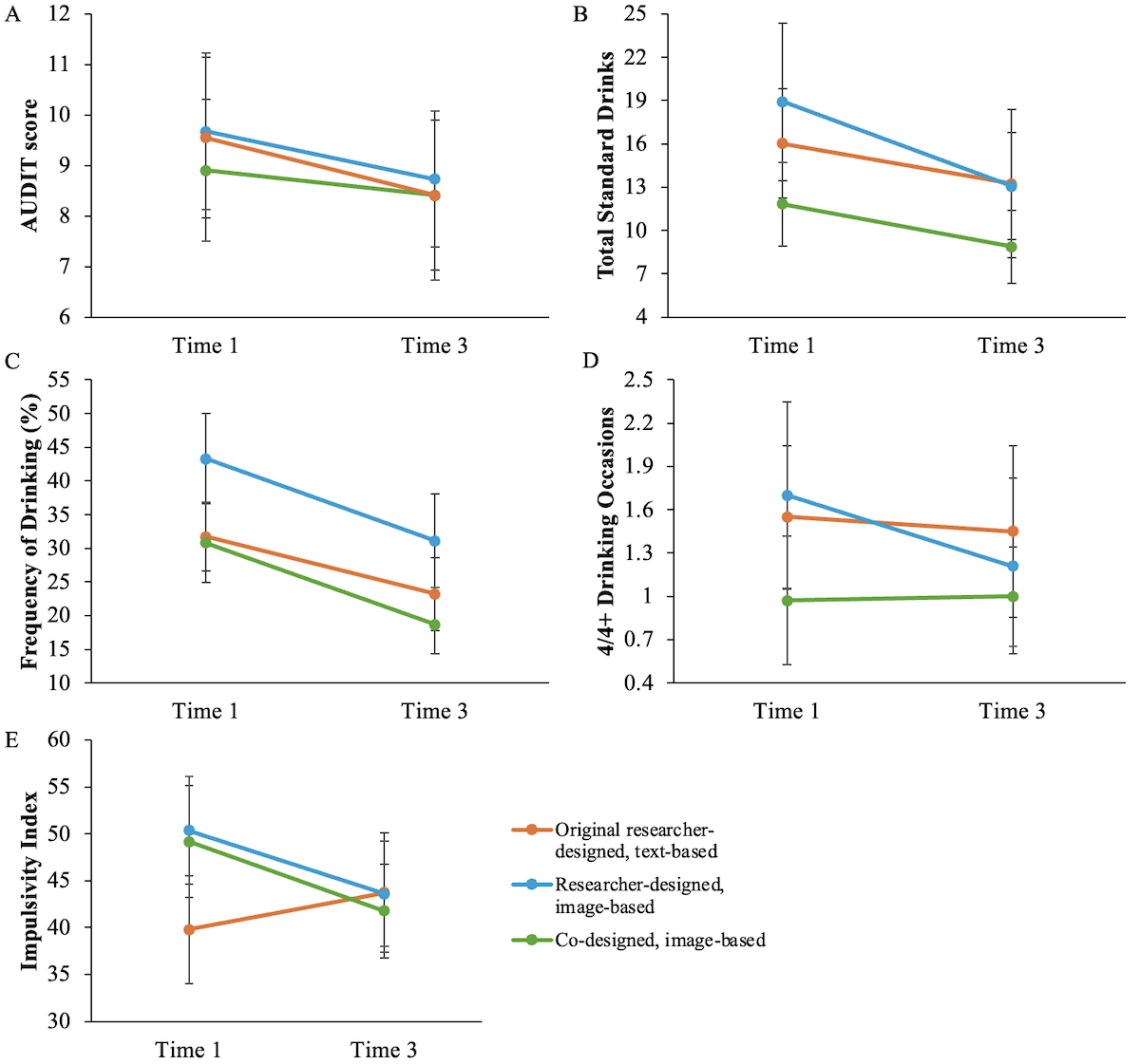
Change in (A) Alcohol Use Disorders Identification Test (AUDIT) score, (B) total standard drink intake, (C) frequency of drinking, (D) occasions on which 4 or more standard drinks were consumed, and (E) impulsivity following personalized feedback regarding alcohol use and impulsivity from T1 to T3 (89.70 days).

With regard to the change in AUDIT scores from T1 (mean 9.32, SD 5.12) to T3 (mean 8.53, SD 5.44), there was a significant main effect of time, *F*_1,88_=4.80; *P*=.03; η_p_^2^=0.05, representing an 8.5% reduction in harm. There was no significant main effect of feedback group allocation, *F*_2,88_=0.08; *P*=.92; η_p_^2^<0.01, and no interaction between time and feedback group, *F*_2,88_=0.24; *P*=.79; η_p_^2^=0.01 (Figure 8A).

Regarding alcohol intake variables derived from Timeline Followback, there was a significant main effect of time, *F*_1,88_=10.98; *P*<.001; η_p_^2^=0.11, on total standard drinks consumed from T1 (mean 17.61, SD 18.60) to T3 (mean 11.78, SD 14.43), representing a 33% reduction in consumption. There was no significant main effect of feedback group allocation, *F*_2,88_=1.31; *P*=.28; η_p_^2^=0.03, and no interaction between time and feedback group, *F*_2,88_=0.75; *P*=.47; η_p_^2^=0.02 (Figure 8B).

There was a significant main effect of time, *F*_1,88_=35.43; *P*<.001; η_p_^2^=0.29, on frequency of drinking from T1 (mean 35.09, SD 21.19) to T3 (mean 24.63, SD 21.68), representing a 30% reduction in drinking occasions. There was a significant main effect of feedback group allocation, *F*_2,88_=3.44; *P*=.04; η_p_^2^=0.07. Pairwise comparisons showed participants in the researcher-designed, image-based (*P*=.002; *d*=0.56) and co-designed, image-based (*P*=.005; *d*=0.56) feedback groups significantly reduced frequency of drinking, whereas those in the original researcher-designed, text-based feedback group (*P*=.15; *d*=0.39) did not. There was no interaction between time and feedback group, *F*_2,88_=0.42; *P*=.66; η_p_^2^=0.01 (Figure 8C).

There was no significant main effect of time, *F*_1,88_=1.31; *P*=.26; η_p_^2^=0.02, on occasions drinking ≥4 drinks from T1 (mean 1.78, SD 2.30) to T3 (mean 1.22, SD 1.97), although there was still a 31.5% reduction in episodes. There was no significant main effect of feedback group allocation, *F*_2,88_=0.74; *P*=.48; η_p_^2^=0.02, and no interaction between time and feedback group, *F*_2,88_=0.96; *P*=.39; η_p_^2^=0.02 (Figures 8D).

#### Change in Impulsivity as a Function of Feedback Group

With regard to the change in average impulsivity index from T1 (mean 46.46, SD 21.72) to T3 (mean 43.12, SD 21.07), there was no significant main effect of time, *F*_1,75_=2.05; *P*=.16; η_p_^2^=0.03, no significant main effect of feedback group allocation, *F*_2,75_=0.44; *P*=.65; η_p_^2^=0.01, and no interaction between time and feedback group, *F*_2,75_=2.84; *P*=.06; η_p_^2^=0.07 (Figure 8E).

## Discussion

### Summary, Implications, and Comparison With the Literature

#### Part 1: Co-Design

In Part 1 of this study, we used a co-design process to develop user-designed personalized feedback about alcohol intake and brain health (especially impulsivity) to incorporate into an existing brief digital intervention that has previously been shown to reduce hazardous drinking [6]. Drawing on research and guidelines that advocate for consumer involvement at multiple stages of the research process [7-9], we consulted with young people when preparing prompts and materials and then conducted a series of focus groups with adolescents and young adults using those same resources.

Following consultation, thematic analysis established that young consumers wanted feedback about alcohol intake and brain health to be visual, clear, accessible, and brief. They disliked wordiness or unduly complicated visual representations. They also wanted more information about how their drinking compared with that of their peers and education regarding the long-term physical and mental health impacts of alcohol use. The text-based researcher-designed feedback used in the original study (original researcher-designed, text-based feedback) as well as the image-orientated researcher-designed feedback developed for use with focus groups (researcher-designed, image-based feedback) already included normative information on how drinking compared with Australian national guidelines. While focus group participants indicated that they wanted this information tailored by age group, evidence suggests that this can lead to increased alcohol consumption among young people who drink below what is considered average for their cohort [61]. We were therefore reluctant to include any peer-specific information that might encourage increased drinking but retained the normative information pertaining to national guidelines.

Recommendations to include information on the long-term physical and mental health impacts of alcohol use were unexpected and revealed genuine interest among our participants in improving education on this front. Attempts to include this level of detail in the co-designed, image-based feedback proved difficult, as the result conflicted with preferences for concise infographics. As such, we determined that this level of detail could be incorporated via links to a (planned) website. At the conclusion of Part 1 of this study, we thus had three versions of brief personalized feedback: (1) original researcher-designed, text-based; (2) researcher-designed, image-based; and (3) co-designed, image-based, suitable for investigations examining preference and efficacy.

#### Part 2: Efficacy

Contrary to our hypothesis, participants who received co-designed, image-based feedback did not score more highly on the COM-B:AIQ, relative to those who received either the original researcher-designed, text-based or researcher-designed, image-based feedback. Groups did not differ in their knowledge of safe drinking levels, their understanding of the impulsivity percentile information, their social and environmental opportunities, or their motivation to change their drinking behavior. Worryingly, irrespective of feedback format, a substantial proportion of the sample remained unaware of guidelines for recommended weekly and maximum daily intake. Large cross-sectional surveys suggest only 32% to 40% of Australians can accurately respond to questions about drinking limits, with younger people demonstrating less understanding than older ones [62]. Several studies have shown even less awareness of daily drinking limits [62,63]. Although all feedback types clearly articulated weekly and daily intake guidelines, the predominance of younger people in our sample may partly account for the relatively low scores on these capacity-based questions. In contrast, and counter to feedback from focus group participants, understanding of percentiles was much higher, although again unrelated to feedback format. As participants were recruited largely from a university student cohort, knowledge related to percentiles might be expected to be higher than in the general population.

As hypothesized, participants preferred the co-designed, image-based feedback over both the original researcher-designed, text-based and the researcher-designed, image-based feedback options. This preference did not vary according to the type of feedback they initially received (as part of investigations into how the different types of feedback impacted consumption) or the order in which they viewed the 3 versions; across all groups, participants rated the co-designed, image-based feedback as having the most appealing look and feel. It is worth noting that no participants involved in Part 1 of this study were recruited into Part 2. Additionally, participants in Part 2 were not informed about how each type of feedback had been generated. Effect sizes indicate that the co-designed feedback was substantially preferred over the researcher-designed, image-based feedback and strongly preferred to the original researcher-designed, text-based feedback. This finding aligns with input from our focus groups, who favored the researcher-designed, image-based feedback over the original researcher-designed, text-based feedback, and is consistent with previous findings that co-designed products are, overall, well-received and well-liked [19,20,64].

Although the co-designed feedback was preferred, there were few significant differences in subsequent alcohol intake behavior as a function of feedback condition. As hypothesized, and replicating the findings of the intervention pilot study [6], there were significant overall reductions in alcohol intake. Across conditions, total standard drinks decreased and there was also a significant reduction in alcohol-related harm. This contributes further evidence for the efficacy of personalized feedback in targeting hazardous drinking, although requires further testing in fully randomized trials. While occasions when 4 or more drinks were consumed in 1 episode decreased across groups, this change was not significant, again aligning with pilot study findings. Also in keeping with the pilot, and as anticipated, there was no change in impulsivity across time.

One group-based difference emerged: participants in researcher-designed, image-based and co-designed, image-based groups reduced their frequency of drinking, whereas those in the researcher-designed, text-based group did not. In the pilot, which used only the original, text-based personalized feedback, there was likewise no significant reduction in drinking frequency. The present findings thus suggest that visual feedback may be more effective than text-based feedback in reducing how often people drink. This is unsurprising given the reactions of focus group participants to the original researcher-designed, text-based feedback and their strong preferences for the visual examples provided. However, this finding does not directly support the greater efficacy of co-designed, image-based feedback compared with that designed without consumer input, as the groups who received researcher-designed, image-based and co-designed, image-based feedback reduced their drinking frequency by the same amount.

### Integration and Future Direction

We used participatory design principles during the preparation phase, throughout the focus groups, and in feedback design. When consulting the Youth Advisory Board at the Matilda Centre, we sought advice on co-design best practice, particularly on facilitating engaging, inclusive, and collaborative focus groups. We aimed to avoid common pitfalls identified in the literature, such as mistaking simple consultation for co-design and limiting participants’ decision-making power or creativity [13]. It is important to note that, although the Youth Advisory Board recommended providing visual feedback examples to the focus groups, they did not contribute to the actual design of the researcher-designed, image-based feedback. This ensured it genuinely reflected our unassisted attempts. Finally, we drew on the recommendations of our focus group participants to develop fully co-designed feedback about alcohol intake, brain health, and how these impact vulnerability for alcohol misuse and alcohol use disorder. Consistent with previous findings, our participants expressed a strong preference for feedback developed via co-design [18-20]. Researchers have noted, however, a paucity of research examining the actual effectiveness of co-designed products and interventions [11,12,25,26]. To address this critical gap, we explored the relative impact of co-designed, image-based feedback across several outcomes, including its influence on participants’ capacity, opportunity, and motivation towards changing their drinking behavior, as well as their subsequent alcohol intake. Notably, co-designed, image-based feedback was no more efficacious than researcher-designed (image- or text-based) feedback in reducing alcohol consumption.

Bevan Jones et al [26] have suggested that future research should explore how academics experience co-design, as most existing work focuses on participant perspectives. Research should consider whether the time, effort, and resources required for co-design are well spent, and how participatory methods can be improved to ensure the process benefits both developers and end users. This study adds a qualifying voice to the widespread, yet largely unevidenced, enthusiasm for co-design. Nonetheless, the improved likeability of our intervention is a valuable outcome of the co-design process: unless people like the look and feel of a product, they may not use it. Although it is less vital in the context of once-off personalized feedback, preference is especially important for products, such as health apps, that people interact with on a repeated and regular basis. Co-design is arguably essential (on both ethical and pragmatic grounds) when designing tools for people with specific conditions and needs or in specialist contexts. Further research is required to guide the incorporation of co-design within specific and varied scenarios, to ensure it adds sufficient value to the end product and to justify its costs in terms of researcher resources.

### Limitations

Some limitations of this study must be noted. Although we endeavored to use participatory principles in Part 1, it is important to recognize that focus groups were supplied with some existing materials. They saw the original text-based personalized feedback as well as examples designed by researchers on the advice of the Youth Advisory Board. This may have influenced their responses and the recommendations they made around the co-designed, image-based feedback. While this challenge is inherent to all participatory processes, future research may seek to address how researchers might tailor studies to maximize participatory input and minimize researcher influence.

In Part 2, returning T2 participants completed the COM-B:AIQ and were then shown all 3 formats of feedback (in counterbalanced order) and asked about preferences. This might have introduced contamination bias and may explain findings at T3 showing participants in all 3 feedback conditions reduced quantity of drinking and harmful alcohol use. Importantly, although participants did see all 3 feedback formats at T2, it was not personalized. No details pertaining to alcohol intake or impulsivity levels were included. Moreover, some condition-based differences were evident at T3. Participants in image-based feedback conditions (researcher-designed and co-designed) reported significant reductions in frequency of drinking, whereas those in the text-based (researcher-designed) condition showed no significant change in drinking frequency. It seems unlikely that having seen all 3 nonpersonalized formats of the feedback had a greater impact on those who received personalized image-based feedback—and only on frequency of drinking—over those who received text-based feedback. Nonetheless, future work will involve a randomized design to minimize the potential for contamination bias.

We did not have a no-feedback control group. As such, we must interpret findings around reductions in alcohol intake behavior and harm cautiously. Nonetheless, there was a control condition in the pilot study, and we saw similar reductions in total alcohol intake, occasions drinking 4 or more drinks in 1 episode, and harm [6]. We also did not ask participants to complete the COM-B:AIQ at T1 (prior to providing feedback) and then again at T2 (following feedback delivery). Thus, we could not explore whether the format of feedback moderated change in capacity, opportunity, or motivation to alter alcohol intake behavior over time. In addition, as with any creative production, it was not possible to consider all the conceivable impacts of different design choices and how these might have influenced the efficacy of co-designed feedback. The same research team developed the co-designed, image-based and researcher-designed, image-based feedback (using only the graphic tools of PowerPoint); thus, the only difference between the 2 types of feedback was input from the focus groups. This was an intentional decision, to control for other factors that could affect the quality of the designs, such as different tools, or designers with varied skill levels. So, although this may have weakened the impact of the co-designed feedback, it demonstrated the influence of stakeholder input on the design process while holding constant other factors.

While our Part 1 sample comprised equal numbers of male and female participants, a higher proportion of female participants undertook Part 2. Recruitment for Parts 1 and 2 involved advertising on social media and via the University of Melbourne student portal. For Part 2, however, students enrolled in first year psychology subjects could secure course credit for participation. Consequently, our Part 2 sample reflects the demographics of this largely female, high school educated cohort; thus, we must be cautious about the generalizability of findings. Female undergraduate students may not be representative of a group motivated to reduce their alcohol consumption. Community or other samples concerned about their alcohol intake may have been more receptive toward the information presented in the feedback. The sample nonetheless did at least drink as much (or more) than a sample drawn from the general population. At T1, 63% (140/222) of our sample reported binge drinking (≥4 drinks per episode) in the past 2 weeks (compared with 24% of Australians who reported binge drinking at least once a month, and 21% of people aged 18-24 years who reported binge drinking at least weekly). Also, at T1, 29% (64/222) of our sample reported drinking more than 10 standard drinks per week on average (compared with 25% of all Australians, and 28% of those aged 18-24 years who reported consuming more than 10 drinks per week on average) [65]. Further, all participants reported being completely honest or mostly honest about their alcohol intake; so, although an undergraduate university sample may not fully represent the wider community, the research is not limited by insufficient alcohol intake levels for reductions to be observed.

Finally, although 75% (166/222) of T1 participants returned for T2 (comparable to the original pilot where 78% returned for T2), only 55% (91/166) of those returned for T3, and the majority were female (75/91, 82%). This level of attrition was partly due to the nature of collecting data from a largely tertiary cohort. T1 and T2 testing took place within the same semester (and completing the protocols was linked to course credit). By contrast, T3 testing occurred in a subsequent semester (and reimbursement was via eGift vouchers). As such, a proportion of students who completed T1 and T2 could have been on leave or would no longer have been enrolled. Such students would not have received the email invite for T3. Others may have been less motivated to continue with the study given participation was no longer linked to course credit. We must therefore be cautious about the generalizability of findings given the level of attrition at T3.

### Conclusions

Using a mixed methods design, we collaborated with consumers to develop co-designed personalized feedback about alcohol intake and brain health to incorporate into an existing brief digital intervention that has previously been shown to successfully reduce hazardous drinking. While participants preferred the co-designed, image-based feedback, it did not appear to influence their capacity, opportunity, or motivation to alter their alcohol consumption behavior. Nonetheless, regardless of the type of feedback participants received, they all significantly reduced their total alcohol intake. There was a significant reduction in harm related to alcohol use and a nonsignificant reduction in binge drinking episodes. Participants in image-based feedback conditions also significantly reduced frequency of drinking. These findings suggest that personalized feedback can be effective in reducing alcohol use and harm even without extensive co-design enhancements. Given co-designed products and interventions require substantial investment of time, resources, and financial commitment, their added value in terms of efficacy should not be assumed. Future research should identify when and for whom co-design yields meaningful gains in effectiveness to ensure the considerable costs associated with this approach are justified and that limited resources are allocated optimally.

## Data Availability

The datasets generated or analyzed in this study are available in the Open Science Framework [66].

## Appendix

Multimedia Appendix 1 Additional data.

## Abbreviations

APM: Advanced Progressive Matrices
AUDIT: Alcohol Use Disorders Identification Test
COM-B: Capacity, Opportunity, Motivation–Behavior
COM-B: AIQ: COM-B: Alcohol Intake Questionnaire
CONSORT: Consolidated Standards of Reporting Trials
COREQ: Consolidated Criteria for Reporting Qualitative Research
GAD-7: Generalized Anxiety Disorder-7
ICJME: International Committee of Medical Journal Editors
MCQ: monetary choice questionnaire
PHQ-9: Patient Health Questionnaire-9
RT: reaction time
SSRT: stop signal reaction time
SST: stop signal task
WHO: World Health Organization
