# Supplementary material for: Evaluating Co-Designed vs Researcher-Driven Personalized Feedback Formats in a Brief Digital Alcohol Use Intervention: Mixed Methods Study
Source: J Med Internet Res. 2026 Jun 12;28:e87393. doi: 10.2196/87393 (PMC13309769; doi:10.2196/87393)
Supplement: Multimedia Appendix 1 [file jmir_v28i1e87393_app1.docx]

**Supplementary Materials**

**Figure S1.** All researcher-designed, image-based examples used as focus group discussion prompts

1. Information about alcohol intake.

(i)


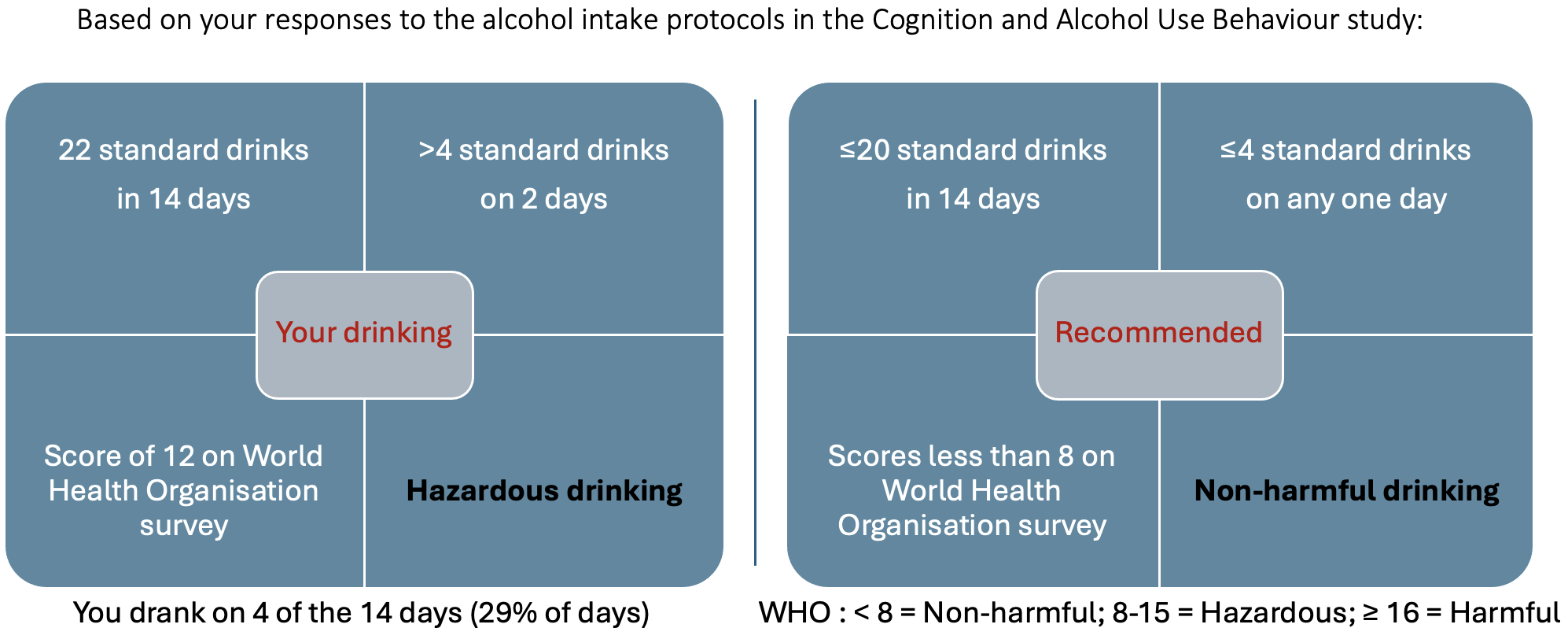


(ii)


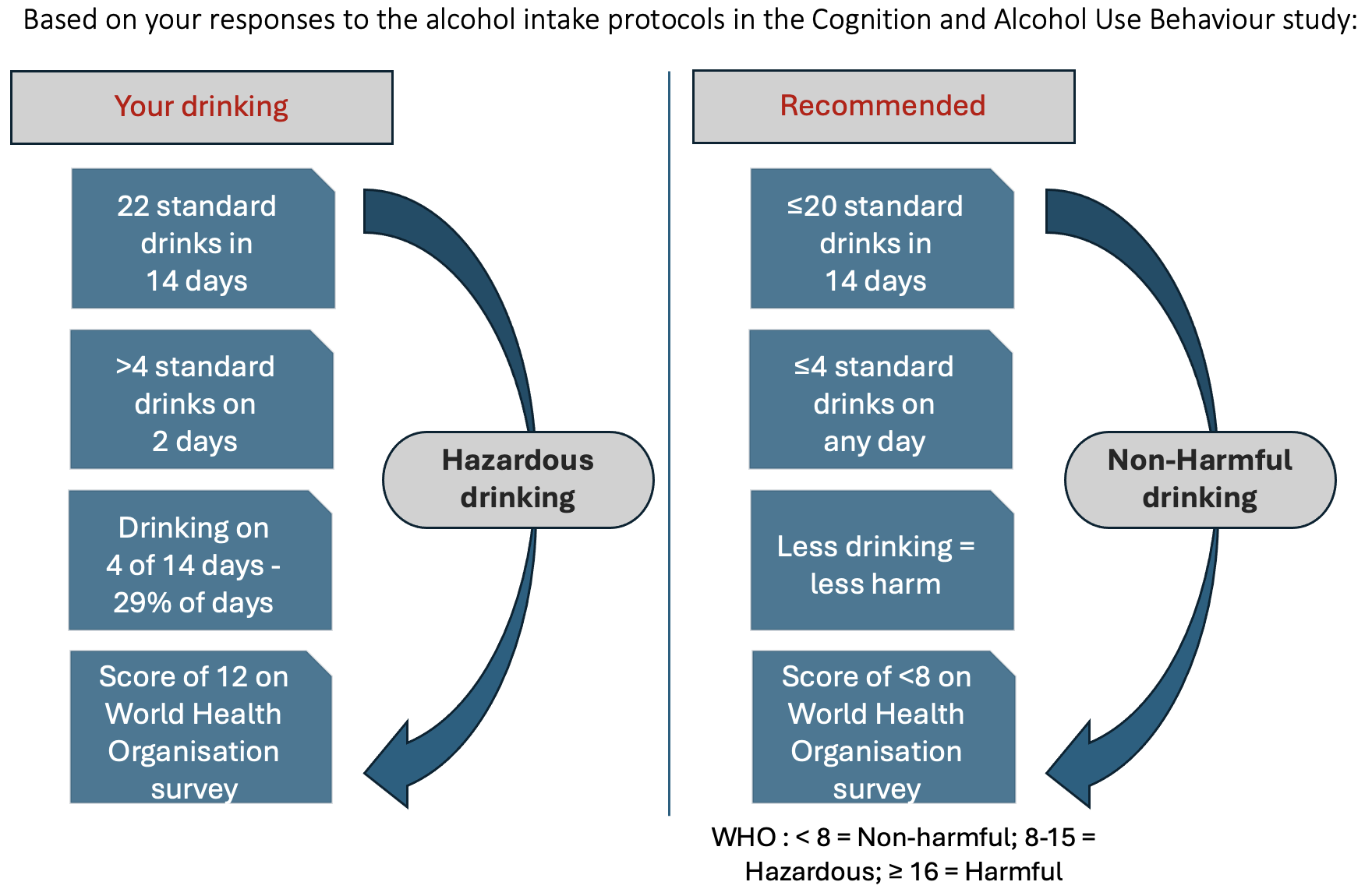


(iii)


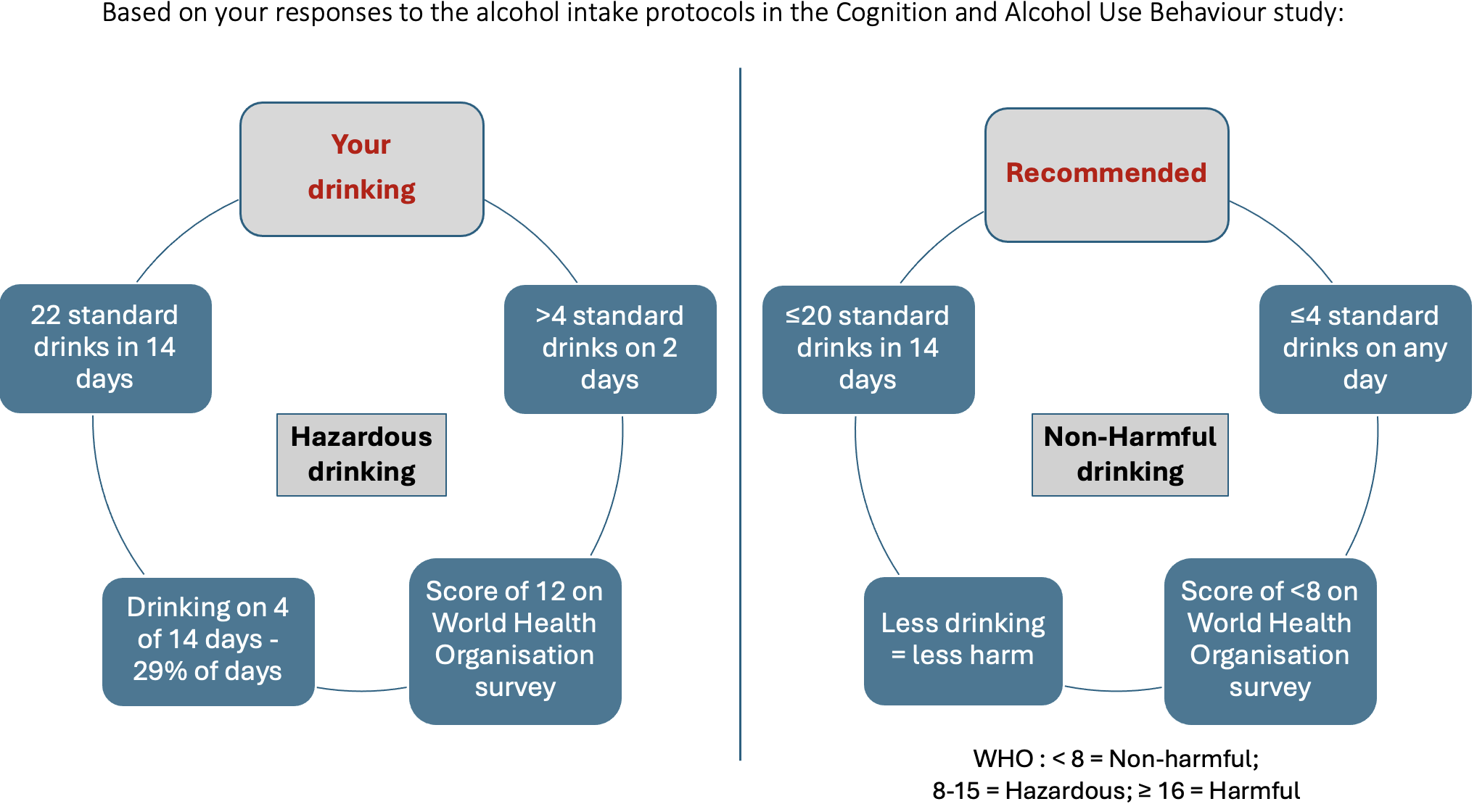


1. Information about impulsivity

(i)


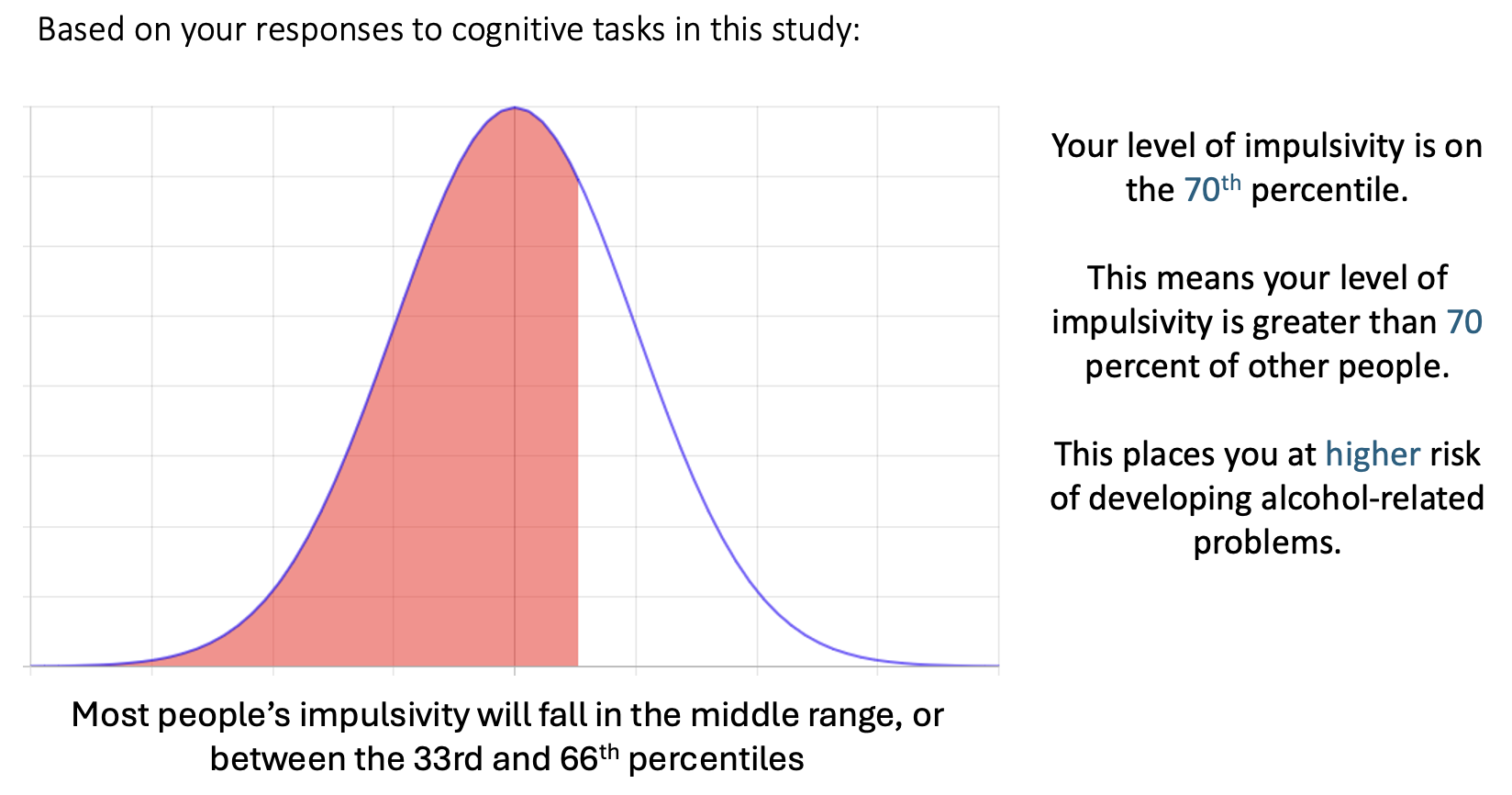


(ii)


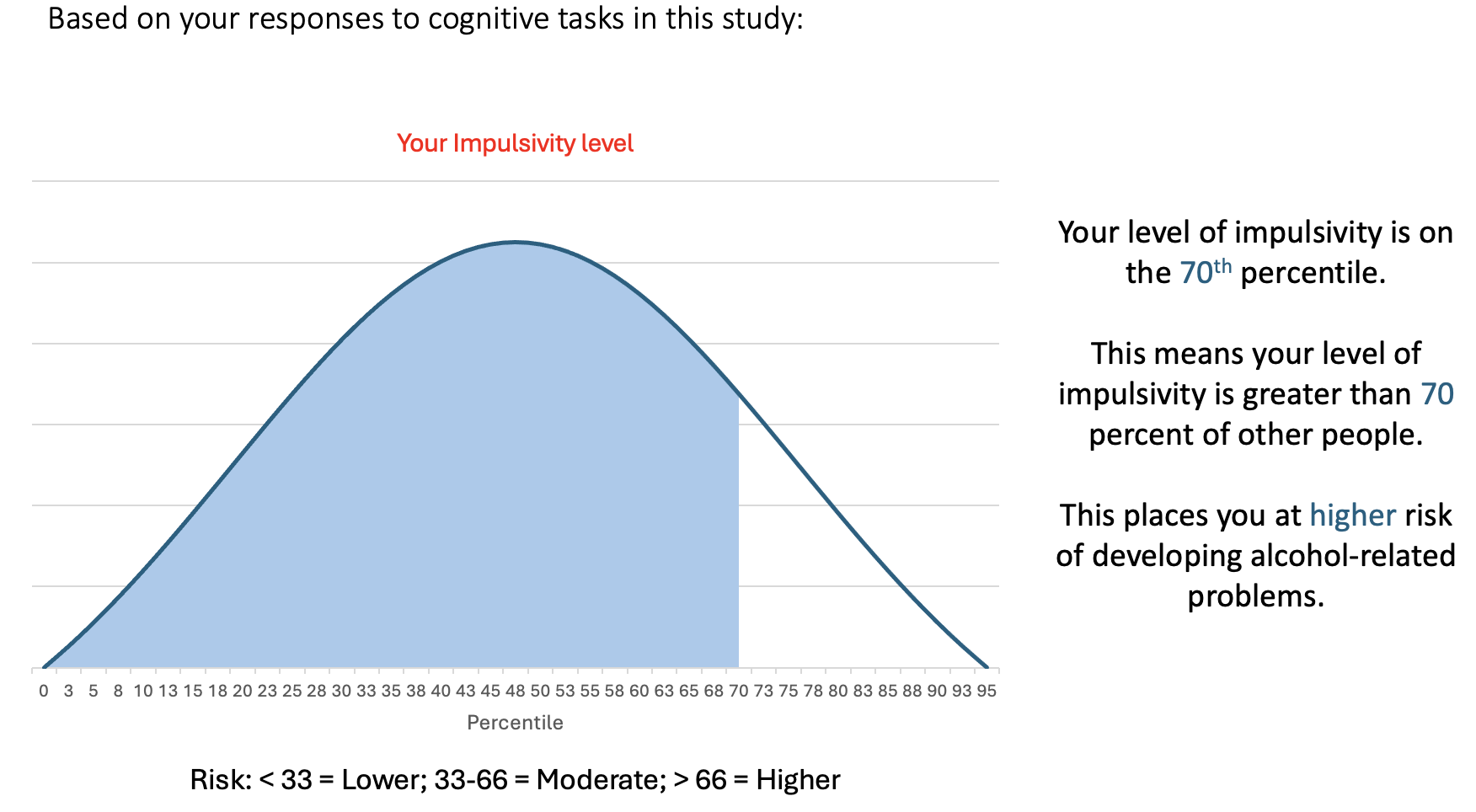


(iii)


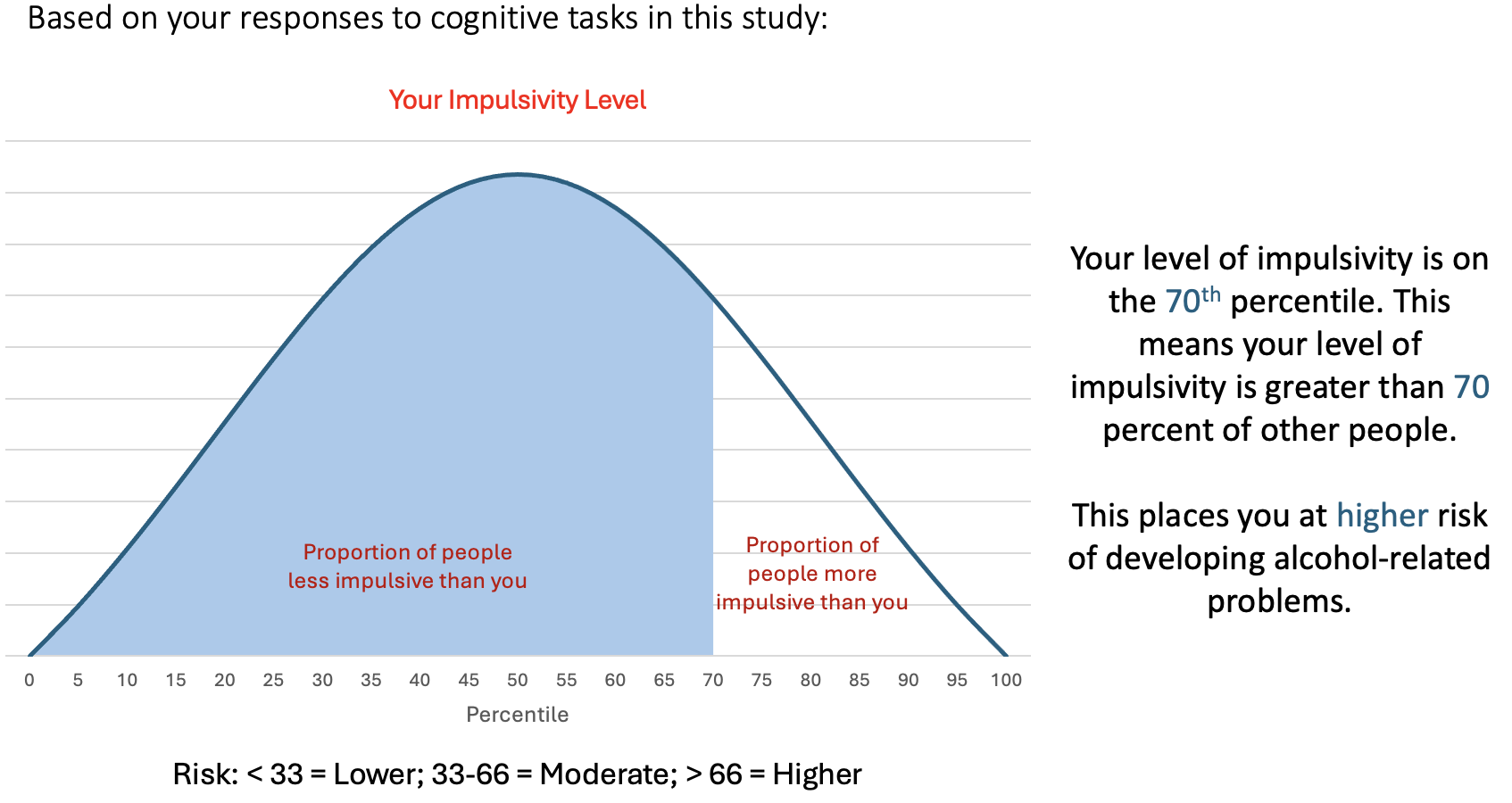


(iv)


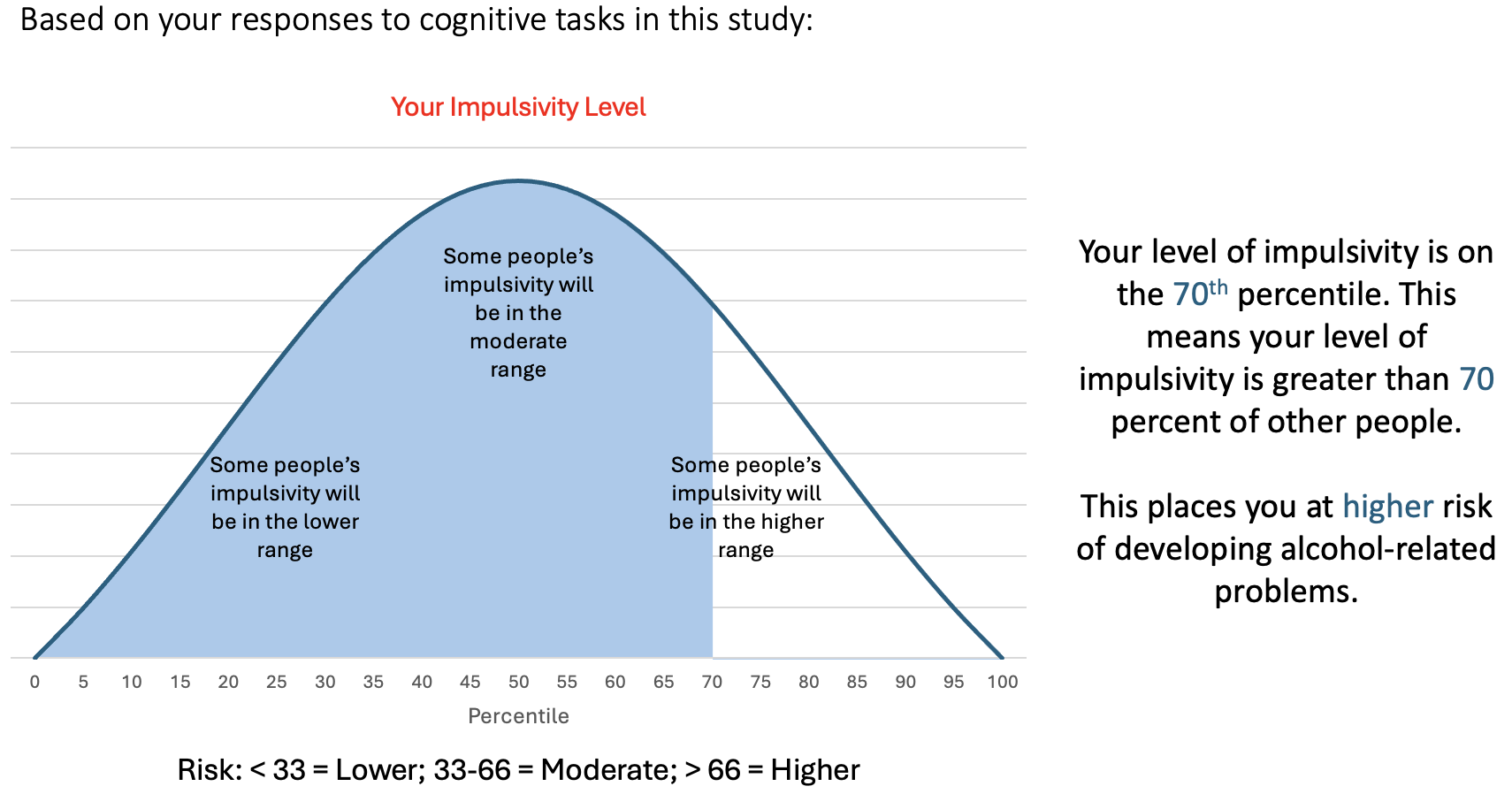


1. General information about the links between impulsivity and alcohol misuse

(i)


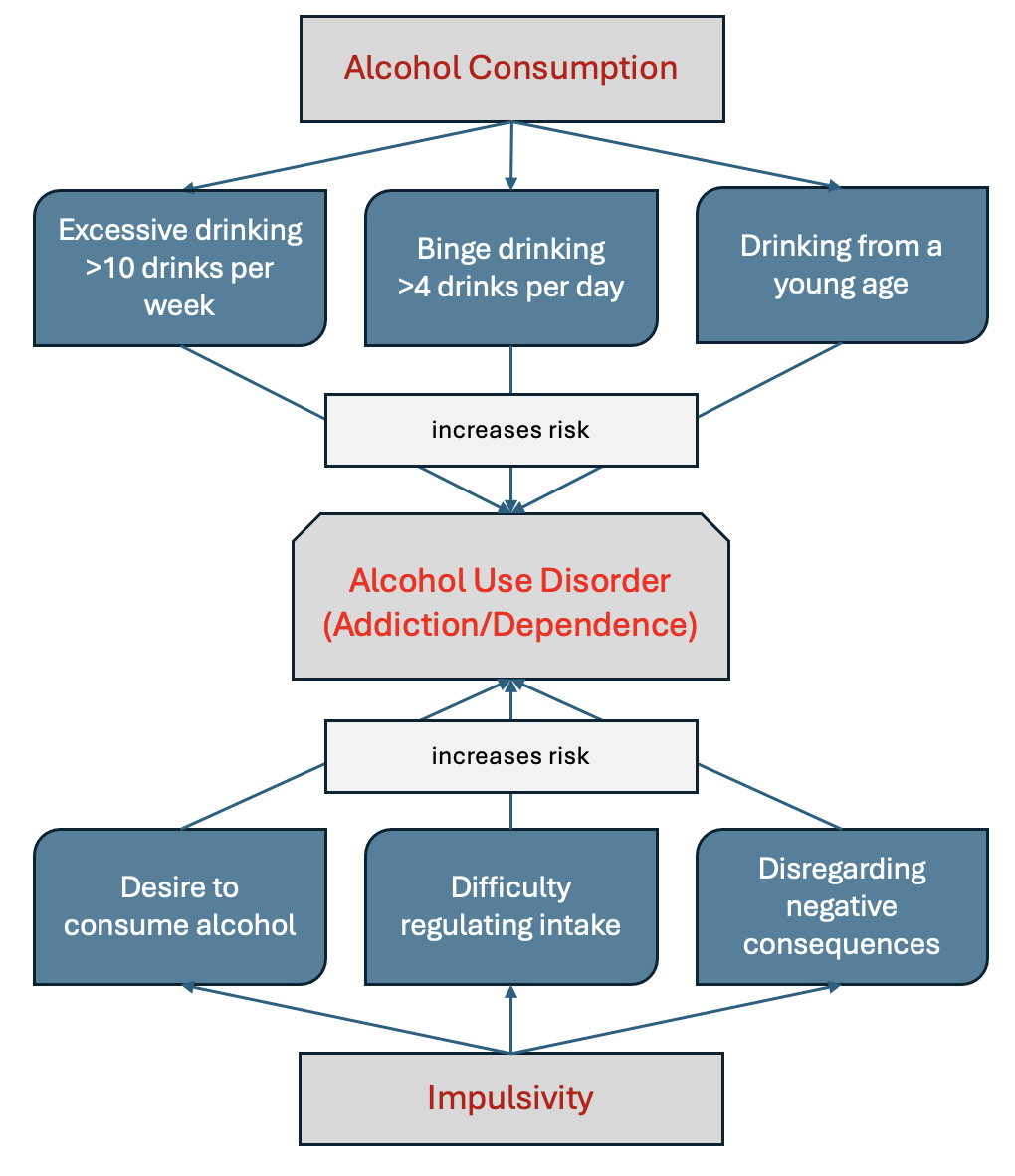


**Table S1.** Consolidated Criteria for Reporting Qualitative Studies Checklist

| **No** | **Item** | **Guide questions/description** |
| --- | --- | --- |
| **Domain 1: Research team and reflexivity** | | |
| **Personal characteristics** | | |
| 1 | Interviewer/facilitator | AP conducted all focus groups with admin support from JB |
| 2 | Credentials | PhD |
| 3 | Occupation | Postdoctoral researcher |
| 4 | Gender | Female |
| 5 | Experience and training | AP is 4 years post PhD with 13 years of experience collecting and analysing research data in tertiary settings. She has served on the University of Melbourne Human Ethics Committee (1 year) and taught undergraduate psychology subjects (13 years). She holds specialised teaching qualifications which enabled her to teach and conduct educational assessments in the secondary sector (15 years). |
| **Relationship with participants** | | |
| 6 | Relationship established | Participants were recruited specifically for this study and not based on a pre-existing relationship with the researchers. At each focus group commencement, AP made casual opening statements designed to establish rapport (e.g., about the weather). |
| 7 | Participant knowledge of the interviewer | Participants were made aware of the study aims via advertisements for the study and a Plain Language Statement disseminated prior to the commencement of focus group sessions. This Statement indicated that AP was one of the lead researchers on the study. At the commencement of focus groups, AP provided details about how the project aligned with her wider research aims (executive dysfunction and alcohol use). |
| 8 | Interviewer characteristics | AP did not provide details about why she is involved in this type of research but did reiterate aims around wanting to improve the format and content of the feedback provided as part of this intervention. |
| **Domain 2: Study design** | | |
| **Theoretical framework** | | |
| 9 | Methodological orientation and theory | No methodological orientation/theory was revealed to participants. Otherwise, the study employed a content analysis framework. |
| **Participant selection** | | |
| 10 | Sampling | Convenience sampling. |
| 11 | Method of approach | Advertisements were posted via the University of Melbourne student portal and other university social media accounts (e.g., Deakin and La Trobe Universities Psychology Student Facebook accounts). Emails were sent to secondary schools inviting psychology teachers to advertise the study to students. Advertisements were posted on the VCE Psychology Teachers Facebook account. Advertisements were also disseminated through local sporting clubs via JB networks (e.g., on Facebook groups including Monash Ultimate Frisbee, Brunswick Ultimate, University of Melbourne Ultimate, Melbourne Beach Ultimate). At each focus group, AP also encouraged participants to pass details of the study to friends. |
| 12 | Sample size | Seven focus groups comprising a total of 40 participants. |
| 13 | Non-participation | Of 42 participants signed-up to attend focus groups, only two failed to attend. No person who commenced participation in a focus group dropped out during the session or requested their data be withdrawn. |
| **Setting** | | |
| 14 | Setting of data collection | Data was collected via Zoom. |
| 15 | Presence of non-participants | No. Participants were asked to attend the session from a quiet location with camera on. |
| 16 | Description of sample | Mean age = 21.87; SD = 7.51; range 17 to 47; 50% female. Twenty-four of the participants were aged under 20 years, 10 were between 20 and 25 years, and six were over 25 years. Average years of education = 14.50 years (SD = 1.85). |
| **Data collection** | | |
| 17 | Interview guide | The Youth Advisory Board at the Matilda Centre for Research in Mental Health and Substance Use (University of Sydney) was consulted for advice on running co-design focus groups that are accessible and inclusive. The Board advised on the design of materials and questions for use in focus group sessions. Members suggested focus groups be presented with an overview of the overarching research, and shown the original researcher-designed, text-based personalised feedback used in the pilot study. They also suggested we develop image-based examples as prompts.  During focus groups, participants were provided with an overview of the broader project. AP then used semi-structured questions to guide discussion but also allowed participants to direct the dialogue where possible. A PowerPoint presentation was used to ensure all groups were exposed to the same materials and questions. Prompts developed for the focus groups are shown in Figure S1. |
| 18 | Repeat interviews | NA |
| 19 | Audio/visual recording | Audio recordings. |
| 20 | Field notes | JB made field notes during sessions where additional contextual or non-verbal information might be missed in an audio recording. |
| 21 | Duration | Focus groups were scheduled for 90-minutes but they uniformly were completed by the 60-minute mark. |
| 22 | Data saturation | We were informed by Hennink et al. (2022) who indicate 4-8 focus groups are required to reach data saturation in qualitative research. We recruited participants and held focus groups until no new data or knowledge was being obtained. |
| 23 | Transcripts returned | No. |
| **Domain 3: Analysis and findings** | | |
| **Data analysis** | | |
| 24 | Number of data coders | Two researchers (AP, JB) independently familiarised themselves with the transcripts, then each generated an initial list of codes, which became a preliminary list of themes. The researchers then collaborated to negotiate a core list of themes, which a third researcher (CC) reviewed against the original transcripts. A final meeting was held with all three researchers, resulting in a consensus list of themes and feedback. |
| 25 | Description of the coding tree | Researchers used Excel to note the preliminary list of themes and to then derive a core list. Participant comments supporting each theme were added below each core item. |
| 26 | Derivation of themes | This study took a strongly inductive, data-driven approach, focusing on what the data said, rather than on a priori theories or conclusions. |
| 27 | Software | Excel spreadsheets. |
| 28 | Participant checking | No. |
| **Reporting** | | |
| 29 | Quotations presented | Participant quotations are presented to illustrate the themes and findings. Quotations are not identified using participant numbers or initials due to the difficulty of identifying distinct speakers in audio recordings. |
| 30 | Data and findings consistent | Yes. |
| 31 | Clarity of major themes | Yes. |
| 32 | Clarity of minor themes | Yes. |

**Table S2.** The COM-B: Alcohol Intake Questionnaire (COM-B:AIQ)

| **Capability** | |
| --- | --- |
| *Knowledge* | |
| 1. What number of standard drinks do you think you can drink in a week on a regular basis before it does significant harm to your health? | |
| 1. What number of standard drinks do you think you can drink on any one day before it does significant harm to your health? | |
| 1. How easy or difficult did you find it to understand the general information provided to you about alcohol use, self-control/impulsivity and links to Alcohol Use Disorder? [1. Extremely difficult to 6. Extremely easy] | |
| 1. How easy or difficult did you find it to understand the **personalised**feedback information provided to you about your level of drinking? [1. Extremely difficult to 6. Extremely easy] | |
| 1. How easy or difficult did you find it to understand the **personalised** feedback information provided to you in percentiles about your level of impulsivity/self-control? [1. Extremely difficult to 6. Extremely easy] | |
| *Skills* |  |
| 1. How often, if at all, do you keep track of how many standard drinks of alcohol you drink each week? [1. Never to 6. Always] | |
| 1. You are told you scored on the 55^th^ percentile in a test. This means:  - You got 55% on the test - 55% of the class scored the same or worse than you - 55% of the class scored the same or better than you - You failed the test - 45% of the class scored worse than you - I don’t know what this means | |
| *Self-efficacy* | |
| 1. How easy or difficult do you find it to drink two or fewer standard drinks of alcohol a day? [1. Extremely difficult to 6. Extremely easy] | |
| 1. How easy or difficult do you generally find it to understand information when it is presented in percentiles? [1. Extremely difficult to 6. Extremely easy] | |
| **Motivation** | |
| *Reflective* | |
| 1. To what extent do you intend keeping your drinking within safe limits? [1. Not at all to 6. Definitely] | |
| 1. To what extent do you believe you will be able to avoid drinking more alcohol than is good for you? [1. Not at all to 6. Definitely] | |
| 1. To what extent do you believe the feedback information provided to you about your level of impulsivity/self-control will assist you to avoid drinking more than is good for you? [1. Not at all to 6. Definitely] | |
| *Automatic* | |
| 1. Nowadays how concerned, if at all, are you about drinking more standard drinks of alcohol than is good for you? [1. Not at all to 6. Definitely] | |
| 1. How concerned, if at all, are you about your level of impulsivity/self-control? [1. Not at all to 6. Definitely] | |
| **Opportunity** | |
| *Social* | |
| 1. How easy or difficult do you think your lifestyle makes it for you to drink less than 10 standard drinks per week? [1. Extremely difficult to 6. Extremely easy] | |
| 1. How easy or difficult do you think your lifestyle makes it for you to drink four or fewer standard drinks of alcohol on any one day? [1. Extremely difficult to 6. Extremely easy] | |
| 1. How easy or difficulty do you think your lifestyle makes it for you to consider your level of impulsivity/self-control when drinking alcohol? [1. Extremely difficult to 6. Extremely easy] | |
| *Environmental* | |
| 1. Do you know where to go if you wanted advice or information on how to cut down on your drinking? [1. No idea to 6. Yes, definitely] | |
| 1. Do you know where to go if you wanted advice or information on impulsivity and self-control? [1. No idea to 6. Yes, definitely] | |

**Table S3.** Consolidated Standard of Reporting Trails (CONSORT) checklist

| **Section / Topic** | **No** | **CONSORT 2025 checklist item description** | **Reported on page no.** |
| --- | --- | --- | --- |
| **Title and abstract** | | |  |
| Title and structured abstract | 1a | Identification as a randomised trial | Not a randomised trial |
|  | 1b | Structured summary of the trial design, methods, results, and conclusions | Figures 6,7; pp.9-10, 18-25 |
| **Open science** | | |  |
| Trial registration | 2 | Name of trial registry, identifying number (with URL) and date of registration | p.15 |
| Protocol and statistical analysis plan | 3 | Where the trial protocol and statistical analysis plan can be accessed | p.15 |
| Data sharing | 4 | Where and how the individual de-identified participant data (including data dictionary), statistical code and any other materials can be accessed | p.35 |
| Funding and conflicts of interest | 5a | Sources of funding and other support (e.g., supply of drugs), and role of funders in the design, conduct, analysis and reporting of the trial | p.35 |
|  | 5b | Financial and other conflicts of interest of the manuscript authors | p.35 |
| **Introduction** | | |  |
| Background and rationale | 6 | Scientific background and rationale | pp.4-5 |
| Objectives | 7 | Specific objectives related to benefits and harms | p.6 |
| **Methods** | | |  |
| Patient and public involvement | 8 | Details of patient or public involvement in the design, conduct and reporting of the trial | pp.6-8 |
| Trial design | 9 | Description of trial design including type of trial (e.g., parallel group, crossover), allocation ratio, and framework (e.g., superiority, equivalence, non-inferiority, exploratory) | pp.9-10 |
| Changes to trial protocol | 10 | Important changes to the trial after it commenced including any outcomes or analyses that were not prespecified, with reason | NA |
| Trial setting | 11 | Settings (e.g., community, hospital) and locations (e.g., countries, sites) where the trial was conducted | p.7,9 |
| Eligibility criteria | 12a | Eligibility criteria for participants | p.7,9 |
|  | 12b | If applicable, eligibility criteria for sites and for individuals delivering the interventions (e.g., surgeons, physiotherapists) | NA |
| Intervention and comparator | 13 | Intervention and comparator with sufficient details to allow replication. If relevant, where additional materials describing the intervention and comparator (e.g., intervention manual) can be accessed | No intervention but see pp.8,9-10 & Figures 1-5 |
| Outcomes | 14 | Pre-specified primary and secondary outcomes, including the specific measurement variable (e.g., systolic blood pressure), analysis metric (e.g., change from baseline, final value, time to event), method of aggregation (e.g., median, proportion), and time point for each outcome | 1. Outcome: co-designed, image-based feedback (qualitative thematic analysis). pp.7-8;15-18 2. Outcome: differences in scores on COM-B:AIQ survey at T2 as a function of feedback type received at T1 (significance level derived from one-way ANOVA analyses). pp.10,13-14,21-23 3. Outcome: scores on preference survey conducted at T2 as a function of feedback type and feedback allocation at T1 (significance level derived from mixed ANOVA analyses and post hoc tests) pp.10,13-14,23 4. Outcome: change in AUDIT score, total standard drinks, frequency of drinking, occasions when ≥4 drinks were consumed in one episode from T1 to T3 (3 months) as a function of feedback type received at T1. pp.10,13-14,24-25 |
| Harms | 15 | How harms were defined and assessed (e.g., systematically, non-systematically) | NA |
| Sample size | 16a | How sample size was determined, including all assumptions supporting the sample size calculation | p.14 |
|  | 16b | Explanation of any interim analyses and stopping guidelines | NA |
| Randomisation: |  |  |  |
| Sequence generation | 17a | Who generated the random allocation sequence and the method used | pp.9-10 |
|  | 17b | Type of randomisation and details of any restriction (e.g., stratification, blocking and block size) | pp.9-10 |
| Allocation concealment mechanism | 18 | Mechanism used to implement the random allocation sequence (e.g., central computer/telephone; sequentially numbered, opaque, sealed containers), describing any steps to conceal the sequence until interventions were assigned | pp.9-10 |
| Implementation | 19 | Whether the personnel who enrolled and those who assigned participants to the interventions had access to the random allocation sequence | pp.9-10 |
| Blinding | 20a | Who was blinded after assignment to interventions (e.g., participants, care providers, outcome assessors, data analysts) | pp.9-10 |
|  | 20b | If blinded, how blinding was achieved and description of the similarity of interventions | pp.9-10 |
| Statistical methods | 21a | Statistical methods used to compare groups for primary and secondary outcomes, including harms | pp.8-9,13-14 |
|  | 21b | Definition of who is included in each analysis (e.g., all randomised participants), and in which group | pp.15,18 |
|  | 21c | How missing data were handled in the analysis | pp.15,18; Figures 6 and 7 |
|  | 21d | Methods for any additional analyses (e.g., subgroup and sensitivity analyses), distinguishing prespecified from post-hoc | pp.15,18 |
| **Results** | | |  |
| Participant flow, including flow diagram | 22a | For each group, the numbers of participants who were randomly assigned, received intended intervention, and were analysed for the primary outcome | Figures 6 and 7; pp. 18,24 |
|  | 22b | For each group, losses and exclusions after randomisation, together with reasons | Figures 6 and 7; pp. 18,24 |
| Recruitment | 23a | Dates defining the periods of recruitment and follow-up for outcomes of benefits and harms | Figures 6 and 7; pp. 18,24 |
|  | 23b | If relevant, why the trial ended or was stopped | NA |
| Intervention and comparator delivery | 24a | Intervention and comparator as they were actually administered (e.g., where appropriate, who delivered the intervention/comparator, how participants adhered, whether they were delivered as intended [fidelity]) | No intervention but see pp.8,9-10 & Figures 1-5 |
|  | 24b | Concomitant care received during the trial for each group | NA |
| Baseline data | 25 | A table showing baseline demographic and clinical characteristics for each group | Tables 1-3 |
| Numbers analysed,  outcomes and estimation | 26 | For each primary and secondary outcome, by group:   - the number of participants included in the analysis - the number of participants with available data at the outcome time point - result for each group, and the estimated effect size and its precision (such as 95% confidence interval) - for binary outcomes, presentation of both absolute and relative effect size | pp.21-25 |
| Harms | 27 | All harms or unintended events in each group | NA |
| Ancillary analyses | 28 | Any other analyses performed, including subgroup and sensitivity analyses, distinguishing pre-specified from post-hoc | NA |
| **Discussion** | | |  |
| Interpretation | 29 | Interpretation consistent with results, balancing benefits and harms, and considering other relevant evidence | pp.25-26 |
| Limitations | 30 | Trial limitations, addressing sources of potential bias, imprecision, generalisability, and, if relevant, multiplicity of analyses | pp.30-33 |

**Table S4.** Specific suggestions for elements to include in the co-designed feedback

| **Information about alcohol intake** | **Information about brain health, esp. impulsivity** | **General information linking impulsivity and alcohol misuse** |
| --- | --- | --- |
| Avoid using < > symbols | Avoid using < > symbols | Use a Venn diagram |
| Use drink icons to show number of drinks | Make sure colours are contrasting and clear | Use active voice |
| Colour days on calendar for drinking days | Even if the comparison is confronting, that's OK | Simplify language. Less academic |
| Use a coloured key for 'hazardous' drinking labels and place close to score; use traffic light colouring | Might be confronting but that's what we want if people are to change their behaviour | Perhaps just use the text.  Having visuals in the previous sections might help balance it |
| Beer glasses as visual to represent number of drinks | Have very clear indicator 'you are here' with arrow and red letters | Use a tree diagram, two branches on top leading to the trunk of AUD |
| Put in dot point format | Split graph into low, med, high, use a key | Make dot points |
| Have side by side bar chart with 'your drinking' and 'recommended' so very easy to compare | Make it even clearer where person stands by adding a 'you are here' mark and arrow – high agreement | Use colours or bold text for keywords and key statistics |
| Emphasise drinking above the recommend, use bolding and red text | Colour code the levels of risk to make it very clear; keep the axis info too, just make it doubly clear the risk levels | Make the graphic horizontal; vertical presentation is "too much like a flow chart" but it doesn't actually flow |
| Use a dial to show going over recommended drinks | Dot point the info on the graph then have matching text colour to the factoids | Visuals would help break up dense text |
| Needs graphics to grab attention and balance text | Prefers the wording 'is **related** to higher risk' rather than ‘**places** you at higher risk’ | “It’s not clear when you first see it, what exactly is contributing to what” |
| Use different font, brighter colours to make it look more interesting | Use confronting images (like in tobacco campaigns) |  |
| Use pie chart for 29% days drinking | Bold the key number (impulsivity score) |  |
